# Single‐cell immune profiling reveals markers of emergency myelopoiesis that distinguish severe from mild respiratory syncytial virus disease in infants

**DOI:** 10.1002/ctm2.1507

**Published:** 2023-12-19

**Authors:** Nevena Zivanovic, Deniz Öner, Yann Abraham, Joseph McGinley, Simon B. Drysdale, Joanne G. Wildenbeest, Marjolein Crabbe, Greet Vanhoof, Kim Thys, Ryan S. Thwaites, Hannah Robinson, Louis Bont, Peter J. M. Openshaw, Federico Martinón‐Torres, RESCEU Investigators, Andrew J. Pollard, Jeroen Aerssens

**Affiliations:** ^1^ Discovery Sciences & Translational Biomarkers Infectious Diseases Janssen Research and Development Beerse Belgium; ^2^ Department of Paediatrics Oxford Vaccine Group, NIHR Oxford Biomedical Research Centre, University of Oxford London UK; ^3^ Centre for Neonatal and Paediatric Infection, Institute for Infection and Immunity, St George's, University of London London UK; ^4^ Department of Pediatric Infectious Diseases and Immunology Wilhelmina Children's Hospital, University Medical Center Utrecht Utrecht The Netherlands; ^5^ Department of Respiratory Medicine National Heart and Lung Institute, Imperial College London London UK; ^6^ Pediatrics Department Translational Pediatrics and Infectious Diseases, Hospital Clínico Universitario de Santiago de Compostela, Santiago de Compostela Galicia Spain; ^7^ Genetics, Vaccines and Infections Research Group (GENVIP), Instituto de Investigación Sanitaria de Santiago, University of Santiago de Compostela Galicia Spain; ^8^ Centro de Investigación Biomédica en Red de Enfermedades Respiratorias (CIBERES), Instituto de Salud Carlos III Madrid Spain

## Abstract

Whereas most infants infected with respiratory syncytial virus (RSV) show no or only mild symptoms, an estimated 3 million children under five are hospitalized annually due to RSV disease. This study aimed to investigate biological mechanisms and associated biomarkers underlying RSV disease heterogeneity in young infants, enabling the potential to objectively categorize RSV‐infected infants according to their medical needs. Immunophenotypic and functional profiling demonstrated the emergence of immature and progenitor‐like neutrophils, proliferative monocytes (HLA‐DR^Low^, Ki67+), impaired antigen‐presenting function, downregulation of T cell response and low abundance of HLA‐DR^Low^ B cells in severe RSV disease. HLA‐DR^Low^ monocytes were found as a hallmark of RSV‐infected infants requiring hospitalization. Complementary transcriptomics identified genes associated with disease severity and pointed to the emergency myelopoiesis response. These results shed new light on mechanisms underlying the pathogenesis and development of severe RSV disease and identified potential new candidate biomarkers for patient stratification.

## Graphical abstract

Single‐cell immune cell profiling reveals emergency myelopoiesis as a hallmark for infants suffering from severe respiratory syncytial virus (RSV) disease. Proliferative HLA‐DR^Low^ monocyte subsets distinguish infants with severe RSV disease. Blood transcriptomic analysis also points to emergency myelopoiesis associated with severe RSV disease and delivers candidate biomarker genes for the stratification of infants with varying RSV disease severity.

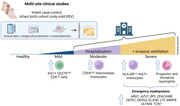

## 1 BACKGROUND

Respiratory syncytial virus (RSV) infection is the leading global cause of hospitalization and mortality in infants with severe acute respiratory tract infections (ARTI).^1^, ^2^, ^3^ Globally, RSV infection resulted in 33.1 million episodes, 3.2 million hospitalizations and 59,600 in‐hospital deaths in children younger than 5 years old in 2015.^3^

About 45% of hospitalizations and in‐hospital deaths occur in infants younger than 6 months old.^3^, ^4^ Premature birth, chronic lung disease, congenital heart disease and immunodeficiency are associated with an increased risk for severe bronchiolitis.^5^ Nevertheless, most children hospitalized with RSV disease are born at term and have no pre‐existing comorbidities.^6^ In severe RSV disease there is a dysregulated immune response as evidenced by an increase in the inflammatory cytokines interleukin (IL)‐6 and IL‐8 in airway fluids^7^, ^8^, ^9^, ^10^ and blood,^11^, ^12^, ^13^ pronounced monocytes (CD69+), T cells (CD8+ T cells and CD3+ double‐negative T cells) and neutrophils,^14^ and decrease of IFN‐y response both in airways^15^, ^16^ and in the blood.^11^, ^17^, ^18^ Mejias et al. used whole blood transcriptomic analyses to attempt to identify biomarkers by translating the abundance or depletion of cell subsets into alterations in gene expression markers.^19^ Whole‐blood transcriptomic analyses also revealed that gene expression signatures such as *MMP8*, *OLFM4* and *ARG1* in severe RSV disease strongly suggest activation of innate immune cell subsets, including monocytes and neutrophils.^20^, ^21^, ^22^ In the study by Heinonen et al., infants aged 6–12 months with mild RSV disease, who were treated as outpatients, demonstrated a higher expression of genes that are related to interferon (IFN) compared to those admitted as inpatients.^23^ This suggests that these older infants had a more robust antiviral response against RSV infection.^23^ Integration of transcriptome and blood immune‐profiling using a 32‐marker panel showed that IFN overexpression is associated with decreased odds of hospitalization, whereas increased numbers of HLA‐DR^Low^ monocytes are associated with increased risk of hospitalization compared with healthy controls.^23^ However, our understanding of pathogenesis based on comprehensive immune profiles is incomplete and clinically useful markers that discriminate between mild, moderate and severe RSV disease and which predict the future course of events are lacking.

One objective of the European Commission‐funded REspiratory Syncytial virus Consortium of EUrope (RESCEU; https://resc‐eu.org) is to identify biomarkers of RSV disease severity in infants to aid clinical management. Whole blood samples were collected from the multinational RESCEU infant case‐control study^24^ and RESCEU birth cohort study.^25^ We investigated single‐cell immune profiles in whole blood of infants with various clinical manifestations of RSV infection using a novel 42‐marker CyTOF panel, allowing simultaneous exploration of cells that carry out innate and adaptive immune responses, including B cell, T cell, monocyte and neutrophil cell subsets. Using this approach, we found evidence of emergency myelopoiesis and dysregulated adaptive immune responses in severe RSV disease compared with mild RSV disease. Random forest analysis identified monocytes with an elevated level of cell cycle marker Ki67 and decreased HLA‐DR expression in hospitalized infants as a correlate for RSV disease severity alongside transcriptomic evidence of activated sets of genes that correlated with levels of immune pathology. These findings not only enhance our understanding of the pathogenesis of RSV disease but also may help identify which RSV‐infected infants need close monitoring, support and future‐specific therapeutics.

## 2 METHODS

### 2.1 Clinical cohort studies

#### 2.1.1 Infant case‐control cohort study

The RESCEU case‐control cohort is a multinational, multicenter, observational study (clinical trial registration number: NCT03756766). The study protocol, study objectives, recruitment, eligibility criteria, informed consent, study procedures and data collection have been described previously.^24^ In brief, infants < 12 months old with RSV disease were recruited from the University Medical Center Utrecht (UMCU) in The Netherlands, Hospital Clínico Universitario de Santiago (SERGAS) in Spain, Imperial College (IMPERIAL) National Health Service Trust (NHS) and Oxford University Hospital NHS Trust (OXFORD) in the United Kingdom during the RSV seasons 2017–2018 (season 1), 2018–2019 (season 2) and 2019–2020 (season 3). Healthy controls without underlying comorbidities were recruited outside of the RSV season.

Eligibility criteria included hospitalization for less than 48 h at enrolment or within 96 h of disease onset, no previous receipt of medications to treat RSV infection, no prior exposure to an investigational RSV vaccine or medication, no previous receipt of immunoglobulins or monoclonal antibodies, and had not used montelukast or oral steroids within seven days before enrolment. Infants with co‐morbidities were included in the differential analysis, however, they were not evaluated in this manuscript.

RSV was detected using an RSV point‐of‐care test (POCT) by either a rapid antigen detection test (Alere I) (Alere Inc) or a rapid RSV polymerase chain reaction (PCR) test at the hospital setting, or an RSV PCR test at the laboratory. Convalescence samples were collected 7 ± 1 weeks after a positive RSV diagnostic test result.

#### 2.1.2 Infant birth cohort study

The RESCEU infant birth cohort study is a multinational, multicenter, prospective and observational study (clinical trial registration number: NCT03627572). The study protocol, design, inclusion and exclusion criteria, recruitment and informed consent procedures have been described previously.^25^

Infants were recruited at birth and followed up during their first year of life throughout three subsequent RSV seasons (2017–2020). Recruitment was conducted at five sites of which three sites collected samples at the moment of RSV infection: UMCU in the Netherlands, Hospital Clínico Universitario de Santiago (SERGAS) in Spain, and Oxford University Hospital NHT Trust (OXFORD) in the United Kingdom. Biological samples were collected when RSV‐ARTI was confirmed by the Alere I RSV POCT assay. Convalescence samples were collected 7 ± 1 weeks after a positive RSV POCT test.

### 2.2 Mass cytometry analyses

Mass cytometry (CyTOF) allows for multiparametric analysis of single cells in complex biological systems^26^ using antibodies labelled with isotopically pure metals^27^ and quantified using inductively coupled plasma mass spectrometry. Current antibody labelling approaches allow for the simultaneous profiling of over 50 molecular markers on a single‐cell level. Relative to conceptually similar fluorescence‐based flow cytometry, where a cell sample is treated with a panel of antibodies, each coupled to a different fluorescent dye, the use of metal‐labelled antibodies in mass cytometry increases the number of parameters that can be measured, reduces the overlap between measured channels and eliminates background autofluorescence.^26^

The relatively low throughput in mass cytometry is typically addressed using sample multiplexing by mass‐tagged cellular barcoding^28^, ^29^ that allows simultaneous analysis of multiple samples in a single measurement. This is accomplished by labelling individual samples with a unique combination of metal tags before being combined into a single sample, stained with a single antibody panel and analysed on a mass cytometer in a single run. Using the following analysis, measured cells are assigned to their corresponding source sample based on their unique mass barcode signatures.

#### 2.2.1 Sample collection

Blood samples were fixed in smart tubes according to the manufacturer's protocol and incubated for 10 min at room temperature (RT), and subsequently stored at −80°C until the processing.

#### 2.2.2 Reference sample

To provide quality control for antibody performance and analysis of batch‐related differences, whole blood reference samples were prepared and included in each measured batch. A 30 mL blood from a single healthy donor was obtained from Janssen Pharmaceutica Biobank. To provide a positive control for cell markers targeted by antibody mixes, healthy donor blood was treated in Smart Tubes with 2 μM Janssen internally developed TLR7 agonist for 6 h at 37°C, 5 % CO_2_, and then fixed for 10 min at RT according to manufacturer's protocol, and further stored at −80°C until use.

#### 2.2.3 Sample batch distribution

A total of 219 samples was divided over 12 batches, with each batch containing 17 RSV‐infected and two healthy infant samples. One reference sample was added to each batch to allow monitoring of the batch‐to‐batch variation in median marker expression and cell population proportion assessment. Allocation of the samples over the different batches ensured equal distribution of infants with respect to infant age, gender and ReSVinet score‐based disease severity.^30^ For six donors, duplicate samples were processed and analyzed as indicated.

#### 2.2.4 Sample processing

Purified metal‐conjugated antibodies obtained from Standard Bio Tools or antibodies labelled using the Maxpar Antibody Labeling Kit (Standard Bio Tools) according to the manufacturer's protocol were used for CyTOF staining and analysis. Prior to staining, samples were thawed in a water bath at 10°C for 20 min with sample mixing by gentle tube inversion after 10 min. After thawing, samples were transferred over a 70 μm cell strainer (Falcon; Corning Inc) to 50 mL tubes containing 25 mL of diluted thaw‐lyse solution (Thaw‐Lyse Buffer; Smart Tube Inc) 1:1000 in nuclease‐free water (UltraPure DNase/RNase‐Free Distilled Water; Invitrogen). Smart tubes were washed 2 times using 3 mL of diluted thaw‐ lyse buffer and the wash was collected in the corresponding 50 mL tubes (Falcon). Samples were mixed and incubated in thaw‐lyse buffer for 10 min at RT, and then centrifuged at 600 x g for 6 min at RT. Following centrifugation, supernatants were removed by decantation and cell pellets were resuspended in 25 mL of diluted thaw‐ lyse buffer, incubated for another 10 min at RT and then centrifuged at 600 x g for 6 min at RT. Following treatment with thaw‐ lyse buffer, cells were washed with phosphate‐buffered saline (PBS; Sigma) and then centrifuged at 600 x g for 6 min at RT. After cell pellet washing with PBS, those samples that still contained traces of red blood cells were further incubated for 10 min at RT with 20 mL of 1:5 diluted lyse‐2 buffer (Lyse 2Buffer; Smart tube Inc) in nuclease‐free water. Cell pellets were further resuspended in 10 mL ice‐cold stain buffer (BD Biosciences) and centrifuged at 600 x g for 6 min at RT.

Samples were resuspended in 1.8 mL of stain buffer and counted using cell counting slides (KOVA Glasstic Slide; Thermo Fisher Scientific). Cell suspensions containing 2 million cells from each sample were transferred to a well of a deep‐well block and centrifuged at 1040 x g for 5 min at 4°C. Samples were then permeabilized by resuspension in 1 mL of Barcode Perm 1:10 diluted in Maxpar PBS, and centrifuged at 1040 x g for 5 min at 4°C. Next, samples were resuspended in 400 μL diluted Barcode Perm Buffer (Standard Bio Tools) and barcoded using 50 μL 20‐plex Pd barcoding reagents (Standard Bio Tools) that were first solved in 1:10 diluted Barcode Perm Buffer for 30 min on ice. After the barcoding step, samples were washed once with diluted Barcode Perm buffer, three times with stain buffer and then pooled into a fluorescence‐activated cell sorting tube.

The pooled sample was resuspended with 50 μL of human TruStain FcX (Biolegend) for 15 min (Fc‐fragment blocking) and stained in 300 μL of antibody mix targeting extracellular targets (Table S1), for 40 min on ice. Following antibody staining, the pooled sample was washed twice in ice‐cold Maxpar Cell Staining buffer (Standard Bio Tools), with centrifugation at 800 x g for 5 min at 4°C. The supernatant was removed, and the cell pellet was resuspended in 1 mL BD Phosflow Perm Buffer III (BD Biosciences) cooled at −20°C, and immediately stored at −80°C until the day of analysis on a mass cytometer.

On the day of analysis on the mass cytometer, the stained pooled sample was removed from the −80°C freezer, washed twice with stain buffer and then stained with 300 μL of antibody mix, targeting intracellular markers for 40 min on ice. Following staining, the pooled sample was washed twice with 2 mL stain buffer with centrifugation at 800 x g for 5 min at 4°C. The sample was then incubated for 20 min on ice, in an Ir‐intercalator diluted in stain buffer (0.5 μL in 1 mL stain buffer) to allow staining of the sample DNA, and then washed three times with 2 mL of stain buffer and centrifuged at 800 x g for 5 min at 4°C. Prior to the last wash step, the samples were aliquoted in four separate vials that were, following centrifugation, stored as cell pellets until analysis.

Before the acquisition, the sample was washed twice in Cell Acquisition Solution (Standard Bio Tools), mixed with EQ Four Element Calibration Beads (Standard Bio Tools), filtered through a 40 μm filter (Falcon) and then analyzed on a Helios mass cytometer with an event rate of approximately 300–400 cells/s.

### 2.3 Computational analysis of CyTOF

Data acquired on CyTOF were obtained in fcs file format and de‐barcoded and normalized using CyTOF Software 6.5.358 for Stand‐Alone Processing Workstations (Standard Bio Tools). After de‐barcoding and normalization, samples were loaded to Cytobank^31^ and manually gated. More than 60 million singlet live cells corresponding to granulocytes and peripheral blood mononuclear cells (PBMCs) were analyzed. The dataset was checked for consistency and reliability using marker enrichment modelling^32^ and Hilbert similarity.^33^ Based on these results one batch was excluded. Cells from the quality‐accepted files were clustered using FlowSOM^34^ into 256 clusters that were manually annotated into metaclusters. The Euclidean distance between clusters was computed based on the median signal intensity of markers used in clustering and the resulting distance was used to build a graph. The corresponding Minimum Spanning Tree was projected using the Kamada Kawai algorithm and used to visualize the output of the clustering step. The clusters were once again checked for consistency, and a set of generalized linear mixed models was fitted to the data. We analyzed differences in cluster, metacluster and size of manually‐gated cell populations between the different ReSVinet disease severity groups.^30^ To account for duplicated samples and multiple measurements (multiple sample collection time points) patient IDs were added as random effects to the model. In addition, differences in signal intensity were assessed for the functional markers included in the antibody panel. Changes in cell abundance were estimated relative to singlets or PBMCs. In this study, we considered changes to be significant if they had a false discovery rate (FDR) corrected *p*‐value of less than 0.05. Significant changes were reported accordingly.

Furthermore, to identify coordinated changes in cellular abundance related to disease severity we trained several random forest models^35^, ^36^ based on cluster or metacluster abundance. Parameters were tuned following best practices and results were assessed using a confusion matrix, variable importance and compositional biplots^37^ based on features selected by each model. To account for populations that were not detected in specific samples, we replaced zeros with pseudo counts corresponding to one cell and corrected the remaining values by substracting the pseudo counts so that the composition remains invariant, as suggested in Martin‐Fernandez et al.^38^ We performed the analysis according to standard practice: for either clusters or metaclusters we first estimated the node size and the number of features from the data, then estimated the top features using the minimal depth algorithm. We then trained a random forest to predict hospitalization status from the abundance of selected features, using Out of Bag cross‐validation. Results were assessed by comparing the predicted label to the ground truth using a confusion matrix; we also reported the variable importance for the top features. Finally, we used compositional biplots^37^ to visualize the contributions of the top features to the separation between the different classes.

Overall, we analyzed 184 samples from RSV ARTI and convalescence visits from infants less than 1 year old. The analysis involved six duplicated samples, which were accounted for in the statistical analysis as indicated above. In the downstream analysis, 32 healthy control infants, and RSV visits of 41 non‐hospitalized infants (mild), 46 hospitalized infants not needing mechanical ventilation (moderate) and 18 hospitalized infants needing mechanical ventilation (severe) were contrasted.

### 2.4 Transcriptome analyses

#### 2.4.1 Sample collection and RNA extraction

The whole blood samples were collected in Paxgene tubes (PAXgene Blood RNA Tube, BD) and stored at −80°C until the processing. The RNA was extracted using the QIAsymphony PAXgene Blood RNA Kit (QIAGEN) according to the manufacturer's instructions. After the quality control, the RNA was further processed for Clariom GOScreen microarray (Thermo Fisher).

#### 2.4.2 Labelling with GeneChip Pico Reagent Kit

First‐strand cDNA was synthesized with a combination of a Poly‐dT and random primers containing a 5′‐adaptor sequence. A 3′‐adaptor was added to the single‐stranded cDNA followed by low‐cycle PCR amplification. The cDNA was used as a template for in vitro transcription that produces amplified amounts of antisense mRNA, (cRNA). The cRNA was then used as input for a second round of cDNA synthesis, producing double‐stranded cDNA. After fragmentation, denaturation and end‐labeling the targets are hybridized on the single GO Screen plate, according to the manufacturer's instructions (Thermo Fisher; GeneChip Pico Reagent Kit). Single sample cartridge arrays were stained on a GeneChip Fluidics Station 450 and scanned on a GeneChip scanner 3000 7G while array plates were stained and imaged on the GeneTitan Multi‐Channel Instrument.

#### 2.4.3 Transcriptomics data preprocessing

Microarray data were preprocessed using R, Bioconductor package.^39^, ^40^ The Robust Multi‐Array Average function was used to normalize the raw data.^41^ Outliers were removed from the downstream data analysis based on visual guidance on the principal component spectral map and sample clustering (Pearson correlation with complete linkage).

### 2.5 Computational analysis of transcriptomics data

Whole blood transcriptomics analysis was performed with the samples from 266 RSV‐ARTI visits, 56 healthy controls and 211 RSV convalescence visits.

The weighted gene correlation network analysis (WGCNA) method was used to integrate cell clusters from the single‐cell immune profiling analysis and the clinical characteristics of disease severity with the transcriptomics data. The WGCNA method was used to create data‐driven gene clusters (modules) of highly correlated genes using the transcriptomic data^42^ from the overlapped samples between single‐cell immune profiling and transcriptomics data (*N* = 169). The transcriptomics dataset (*N* = 169) consisted of 69 RSV‐ARTI visits from otherwise healthy infants, 18 RSV‐ARTI visits from infants with any comorbidities, 51 RSV convalescence visit samples and samples from 31 healthy control infants. The analysis was performed with the WGCNA package in R using the unsigned method (both positive and negative correlations were considered). Sample clustering was applied to detect outliers (cut height 120). The minimum cluster size was set to 10. Seven samples were identified as outliers, so 162 subjects were used for the downstream analysis. The modules were created with the minimum module size including a minimum of 25 genes, and soft power 3, which corresponds to the scale‐free topology fit index is 0.8, as advised in the literature.^43^ After the calculation of the network adjacencies, the adjacency matrix was converted to a topological overlap matrix (TOM), and the genes were clustered based on the TOM‐based dissimilarity. Module eigengenes (MEs) were created with a minimum module size of 25 genes. After the creation of MEs, the modules were related to clinical RSV disease severity parameters of RSV‐ARTI visits from otherwise healthy and healthy control infants, and cell count data of the cell clusters generated from the CyTOF analysis.

We investigated the transcriptomics data with unsupervised principal component analysis (PCA) to explore the biggest contributor of the gene expression variation between the samples and supervised differential expression analysis (*limma*) to understand the gene expression alterations by disease severity and their biological implications. With the PCA analysis, we identified the effect of the collection site. This technical effect was corrected using the ComBat function of the sva package in R.^44^ For the supervised differential expression analysis, the limma (linear models for microarray data) package with empirical Bayes linear models was used.^45^ Age groups (0–3 months, 3–6 months, > 6 months) and sex data were added as covariates and the effect of the subjects was blocked. Fold change (FC) and FDRs were used for the identification of significantly differentially expressed genes. Gene ontology (GO) analysis was investigated using the GOstats package in R.^46^, ^47^ A hypergeometric model was used to investigate GO biological terms using significant (FDR *p*‐value < .05) up‐ and down‐regulated genes separately.

Sparse partial least squares discriminant analysis (sPLS‐DA) was conducted using the MixOmics package in R (ver = 6.24). sPLS‐DA was used to compress the data in a supervised manner using severity groups determined from the ReSViNET score. sPLS‐DA allows for the specification of the number of genes to be used in the training of the model. For this analysis, the top 100 genes contributing to components 1 and 2 were used in the training of the model. PLS‐DA analysis aims to find a set of coefficients/weights for features that result in the maximum possible separation between the dependent variable‐based groups in projected space. The loadings/variable importance of genes in this analysis correspond to how strongly they drive the mapping of a specific sample in a specific cardinal direction in the projected data.

## 3 RESULTS

### 3.1 Clinical characteristics of the infant cohorts and strategy for immune profiling and transcriptomics analyses

**FIGURE 1:**
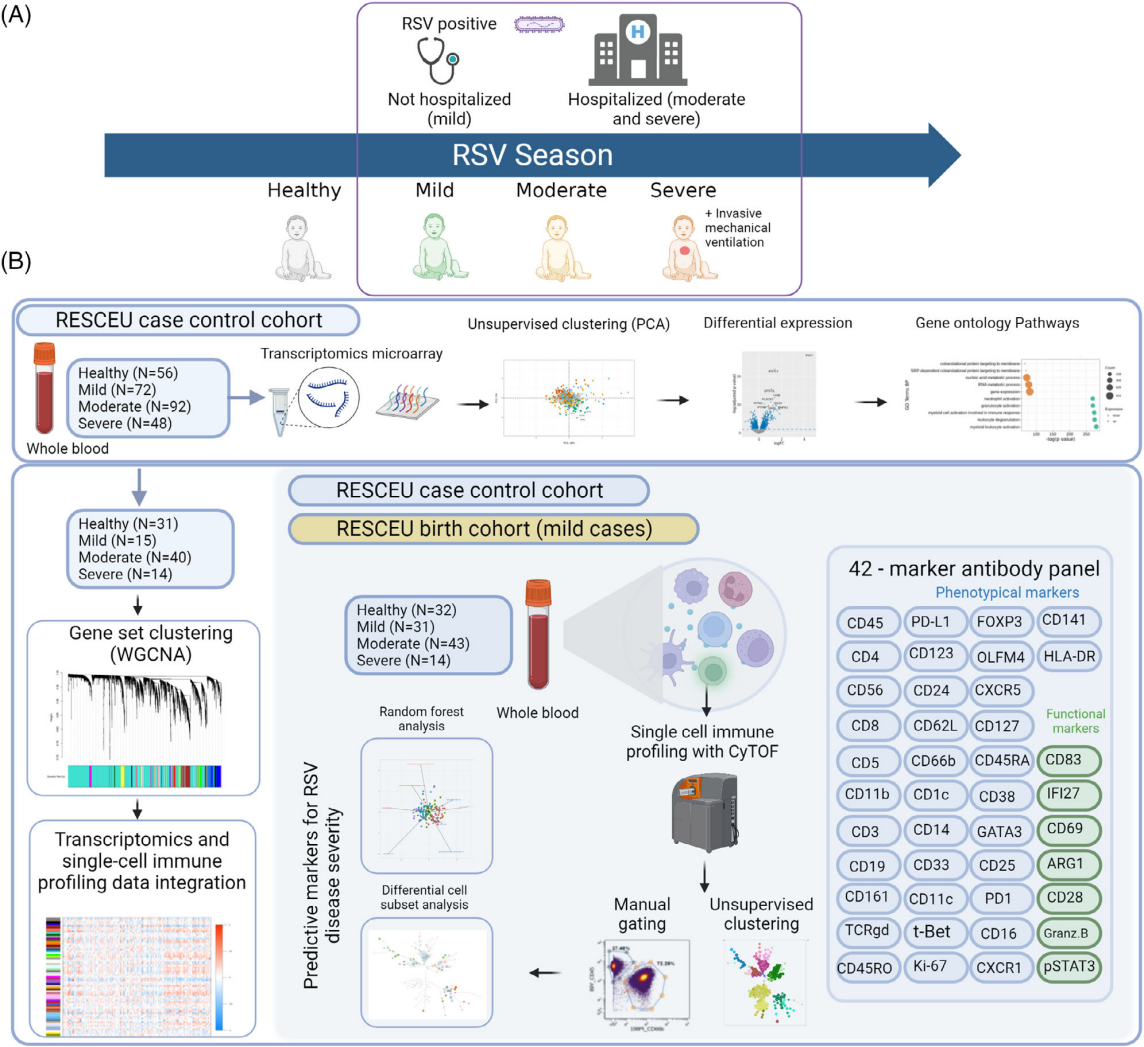
Schematic overview of the cohort and the strategy for single‐cell immune profiling and transcriptomics analysis. (A) Infants with no respiratory syncytial virus (RSV) disease (referred to as healthy) and RSV‐positive infants were recruited from outpatient clinics (mild) and from hospitals (moderate). Infants with severe RSV disease received mechanical ventilation. (B) Whole blood samples were analyzed with a 42‐marker antibody panel enabling profiling of a wide range of cell subsets and search for correlates or predictors of disease severity (experiment 1). Transcriptomics data from the REspiratory Syncytial virus Consortium of EUrope (RESCEU) case‐control study was used to define differential gene expression and gene ontology pathway analysis to understand the transcriptional profile of the disease (experiment 2) and to integrate with the single‐cell immune profiling data to associate a set of gene expressions with cell subsets (experiment 3). Created with BioRender.com.

The RESCEU case‐control study recruited 325 RSV‐infected infants testing positive for RSV either from inpatient or outpatient clinics from the UMCU in the Netherlands, Hospital Clínico Universitario de Santiago (SERGAS) in Spain, Oxford University Hospital NHS Trust (OXFORD) and Imperial College London in the United Kingdom during 2017–2020.^24^, ^48^ The RESCEU birth cohort study was included in the single‐cell immune profiling analysis to complement the number of infants with mild RSV disease. Whole blood samples from 88 RSV‐infected infants with no co‐morbidities and 32 healthy control samples were analyzed for the single‐cell immune profiling study using mass cytometry (CyTOF) to explore the divergent immune response within the hospitalization parameters to measure RSV disease severity (Figure 1). Next, we analyzed whole blood transcriptomics data of 212 RSV‐infected infants with no co‐morbidities, and 56 healthy control samples, to explore differential gene expression profiles within the RSV disease severity groups. Sixty‐nine RSV visits and 31 healthy control samples overlapped between these two analyses. Disease severity groups were classified by hospitalization status: mild RSV disease being non‐hospitalized infants, moderate RSV disease being hospitalization without needing mechanical ventilation and severe RSV disease being needed for mechanical ventilation (SIMV/HFO), and the terms used consistently throughout the manuscript. Using the clinical information about infant hospitalization status, infants with mild and moderate RSV disease were compared with healthy infants as a control group and RSV disease severity was also studied by comparing severe versus mild and moderate RSV disease.

The majority of the infants with severe RSV disease were younger than 3 months old (*N* = 11 (78.6%)), and the majority of infants with mild RSV disease were older than 6 months old (*N* = 20 (64.5%)) (Table 1). The sex of the infants was balanced between the groups. Most infants requiring mechanical ventilation were recruited at the UMCU in the Netherlands. Clinical features in the transcriptomics dataset were similar to the single‐cell immune profiling study in terms of age, sex, timing of sampling and ReSVinet score distribution (Table S2).

**TABLE 1:** Clinical and demographic characteristics of the infants for the single‐cell immune profiling.

		MILD	MODERATE	SEVERE
	Healthy controls	(RSV‐infected, non‐hospitalized)	(RSV‐infected, hospitalized without mechanical ventilation)	(RSV‐infected, hospitalized with mechanical ventilation)
*Infants*	*(N = 32)*	*(N = 31)*	*(N = 43)*	*(N = 14)*
**Age at enrolment (in days)**				
Below 3 months	2 (6.3%)	6 (19.4%)	20 (46.5%)	11 (78.6%)
3–< 6 months	11 (34.4%)	5 (16.1%)	13 (30.2%)	2 (14.3%)
6–12 months	19 (59.4%)	20 (64.5%)	10 (23.3%)	1 (7.1%)
**Age at enrolment (in days) Only infants younger than 6 months**				
Number of infants (N)	13	11	33	13
Mean (SD)	109 (35.6)	88.9 (45.8)	75.9 (46.8)	59.2 (36.1)
Median [Min, Max]	103 [48, 165]	89 [35, 165]	56 [15, 165]	43 [20, 134]
**Sex**				
Female	7 (21.9%)	18 (58.1%)	19 (44.2%)	6 (42.9%)
Male	25 (78.1%)	13 (41.9%)	24 (55.8%)	8 (57.1%)
**Season**				
Season1	0 (0%)	26 (83.9%)	13 (30.2%)	12 (85.7%)
Season2	32 (100%)	3 (9.7%)	26 (60.5%)	2 (14.3%)
Season3	0 (0%)	2 (6.5%)	4 (9.3%)	0 (0%)
**Study**				
Case‐control	32 (100%)	14 (45.2%)	42 (97.7%)	14 (100%)
Birth cohort	0 (0%)	17 (54.8%)	1 (2.3%)	0 (0%)
**Site**				
OXFORD	8 (25.0%)	5 (16.1%)	30 (69.8%)	2 (14.3%)
UMCU	24 (75.0%)	26 (83.9%)	13 (30.2%)	12 (85.7%)
**Gestational Age (in weeks)**				
Mean (SD)	39.6 (0.989)	39.6 (1.12)	38.2 (6.11)	39.1 (1.14)
Median [Min, Max]	40.0 [37, 42]	40.0 [37, 41]	39.0 [0, 42]	39.0 [37, 41]
Missing	1 (3.1%)	0 (0%)	0 (0%)	0 (0%)
**ReSVinet Score**				
Mean (SD)	NA (NA)	3.74 (1.86)	8.63 (3.18)	14.9 (2.54)
Median [Min, Max]	NA [NA, NA]	3.00 [1, 9]	9.00 [3, 15]	15.0 [10, 18]
Missing	NA (NA)	0 (0%)	0 (0%)	0 (0%)
**Time of sampling**				
Mean (SD)	NA (NA)	3.22 (1.58)	4.75 (1.82)	4.33 (0.985)
Median [Min, Max]	NA [NA, NA]	3.00 [1, 7]	5.00 [2, 8]	4.00 [3, 6]
Missing	NA (NA)	4 (12.9%)	31 (72.1%)	2 (14.3%)

Data are represented as the number of samples (%) unless otherwise stated. Mechanical ventilation is defined as the use of invasive ventilation (SIMV/HFO), whereas non‐mechanical ventilation is defined as the use of O_2_ (> 21%), low‐flow nasal cannula, CPAP/BiPAP and high‐flow oxygen therapy. The time of sampling is calculated as the RSV visit date—symptom onset date.

### 3.2 Phenotypic immune profiling of blood from RSV infants

**FIGURE 2:**
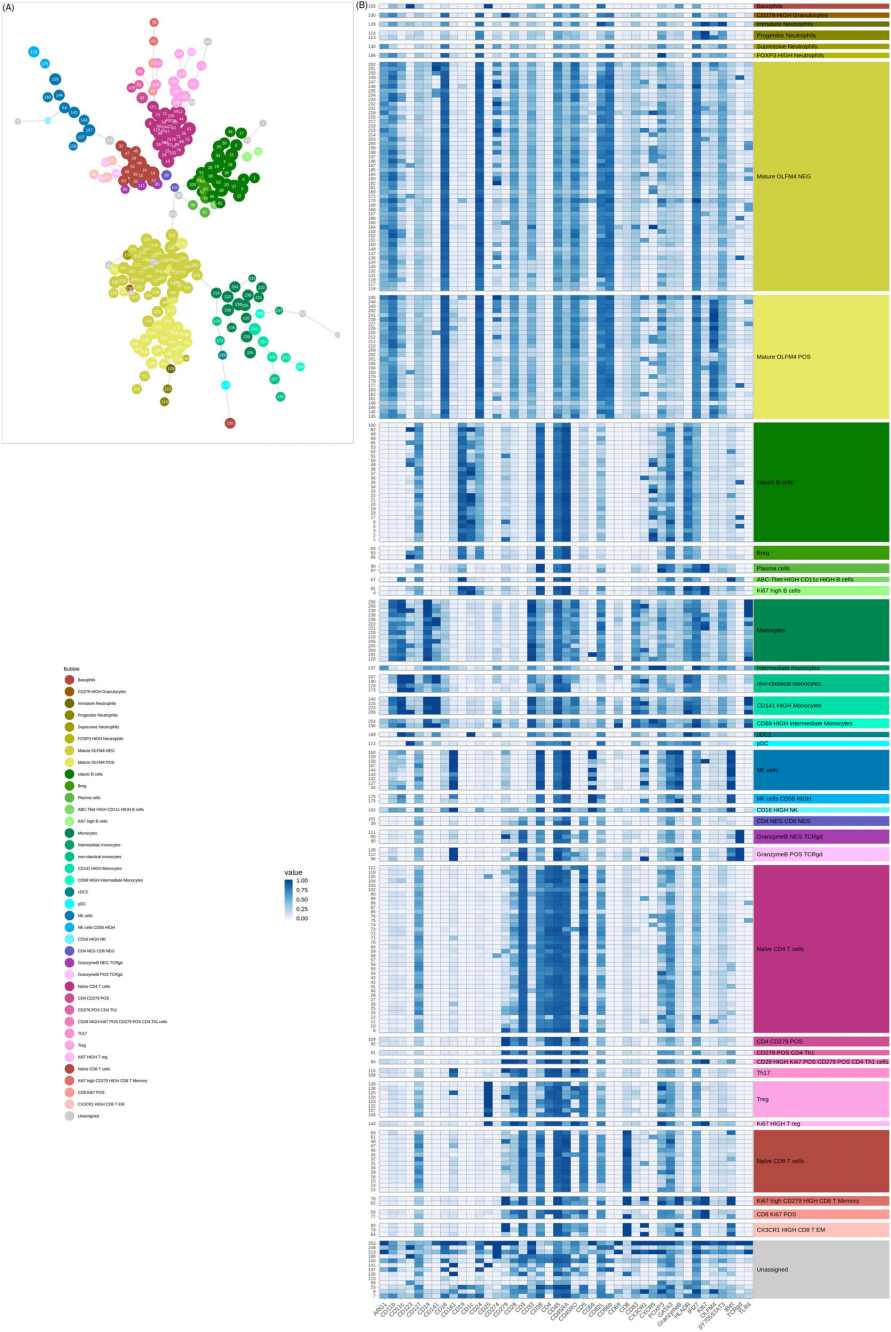
Unsupervised clustering with FlowSOM and cluster‐specific median marker expressions. (A) Minimum spanning tree representation of FlowSOM clusters and metaclusters. Individual cell clusters (circles) are numbered while meta clusters (referred to as ‘bubble’ in the legend) are represented by different colors. (B) Heatmap showing cell clusters grouped by meta cluster and median expression level for each specific marker. Cluster numbers on the y‐axis; marker names on the x‐axis. Meta clusters are sorted by color as represented in the legend. Apart from CD69, CD83, granzyme B, p‐Y705‐STAT3 (‘functional’ markers) and CD127, all measured markers were used for cell subset phenotyping (‘phenotypic’ markers) and used for FlowSOM clustering.

To comprehensively characterize different cell subsets in infant peripheral blood, we designed a 42‐marker antibody panel and performed system‐level blood immune profiling using mass cytometry. FlowSOM unsupervised clustering was applied based on all phenotypic markers (Figure 1). Using a 16 × 16 SOM‐grid, this analysis yielded 256 cell clusters. Clusters were then assigned to 37 metaclusters representing peripheral blood immune cell populations, including T cells, granzyme+ and granzyme– TCRγδ T cells, pDC, cDC2, monocytes, B cells, NK cells, granulocytes, basophils and one metacluster combining clusters with unspecific phenotype. During further analysis of clusters with unspecific phenotype cluster 126 was identified as mucosal‐ associated invariant T (MAIT) cells. FlowSOM clusters were annotated based on median marker expression, as illustrated in Figure 2, and the overlap of cell clusters and metaclusters with manually gated cell populations (Figures S1–S3).

### 3.3 Severe RSV is characterized by a high neutrophil/lymphocyte ratio

**FIGURE 3:**
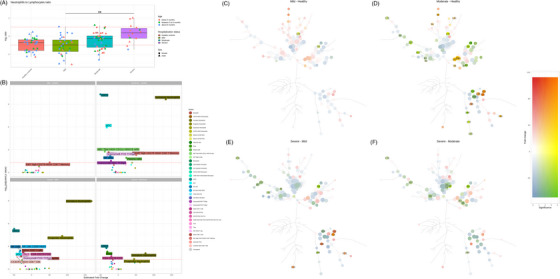
Neutrophil to lymphocyte (N/L) ratio and metaclusters of cells including immature and progenitor neutrophils associated with different degrees of respiratory syncytial virus (RSV) disease severity. (A) Box plot of neutrophil to lymphocyte ratio (log2 ratio) in infants grouped in classes of increasing RSV disease severity (hospitalization status). Age and sex data were plotted with different colours and shapes, respectively. The 2 horizontal lines (red dashed) represent a 2‐fold increase or decrease in the ratio (1 and −1 on log2 scale). (B) Volcano plots show differential analysis of cell subsets for various comparisons of infants with different degrees of RSV disease severity. The estimated fold change is shown on the x‐axis and ‐log10 of the adjusted *p*‐value is shown on the y‐axis. The horizontal lines represent false discovery rate (FDR)‐adjusted *p*‐value < 0.05. (C) Results of differential analysis at the cluster level are visualized as Tree Blend plots: for each cluster 2 colour scales are built corresponding to estimated fold change (blue to red for depleted and enriched clusters respectively) and significance (from white to yellow for the negative log_10_ of the FDR‐adjusted *p‐*value) and mixed to generate a set of colours representative of both variables. For each contrast, the clusters in the Minimum Spanning Tree projection are coloured after the mixed colours corresponding to the results of the significance test. For the tree blend cell cluster legends, please refer to Figure 2A.

For the analysis of the relative abundance of lymphocytes and neutrophil cell subsets, the neutrophil to lymphocyte (N/L) ratio was calculated and contrasted across infant RSV disease groups (Figure 3A). The neutrophil and lymphocyte subsets were determined by aggregating cell clusters belonging to lymphocyte or granulocyte cell subsets. The N/L ratio in infants with mild RSV disease was similar to the ratio in the group of healthy infants. However, the N/L ratio progressively increased in moderate to severe RSV disease, and it was significantly higher in severe versus mild RSV disease (FDR‐adjusted *p*‐value < .05).

### 3.4 Phenotypic immune profiling in relation to RSV disease severity

We contrasted patient groups based on the changes in cell population frequency at the metacluster (‘bubble’) level relative to the total cell number included in the singlets gate (Figure 3B, as defined in manual gating strategy in Figure S1A), focusing on significantly modulated cell metaclusters (FDR‐adjusted *p*‐value < 0.05). We estimated the same contrast including only the infants younger than 6 months old to ensure that our findings were not influenced by differences in age between the groups (Figure S4).

*Mild RSV disease is marked by the cytotoxic T‐cell response*. Relative to healthy infants, mild RSV disease was characterized by an increase in memory CD8 T cell metacluster population, indicating activation of a cytotoxic CD8 T cell‐driven anti‐viral response in both younger (< 6 months old) and all infants (< 1 year old) (Figure 3B and Figure S4, upper left panel). This cell subset showed proliferative capacity based on the high Ki67 levels and high expression of CD279, a marker linked to T cell activation and/or exhaustion.^49^

*Moderate RSV disease presents with a mixed immune response*. Relative to healthy infants, moderate RSV disease showed a lower abundance of antigen‐presenting cell subsets cDC2 and pDC, NK cells, granzyme+ and granzyme– TCRγδ T cells, T‐bet^High^ atypical B cells (ABCs) and basophils in both younger (< 6 months old) and all infants (< 1 year old) (Figure 3B and Figure S4, upper right panel), similar to infants with severe RSV. Conversely, similar to mild RSV disease, moderate RSV disease showed activation of the anti‐viral response indicated by the upregulation of proliferative memory CD8 T cells relative to healthy infants. In addition, higher frequencies of antibody‐producing plasma cells and immature neutrophil subsets were observed in moderate RSV disease relative to healthy infants. Taken together, moderate RSV in infants hospitalized without mechanical ventilation showed a mixed immune response, combining features of activated cellular and humoral immune responses along with a decrease in NK cells and TCRγδ T cells, inflammatory response and myeloid dysregulation.

*Severe RSV disease is marked by decreased T‐cell subsets and immunotolerance with activation of emergency myelopoiesis*. Compared with infants with mild and moderate disease, infants with severe RSV disease showed lower proportions of different T cell subsets, including naïve CD8, cytotoxic CX3CR1+ effector memory (EM) CD8, Th17 CD4, CD279+ CD4 T cells and granzyme B+ TCRγδ T cells in both younger (< 6 months old) and all infants (< 1 year old) (Figure 3B and Figure S4, lower left panel). Furthermore, severe RSV disease was associated with lower proportions of NK cells and CD56^High^ NK cell subsets, basophils and professional antigen‐ presenting cells pDC and cDC2 metaclusters relative to mild disease in both younger (< 6 months old) and all infants (< 1 year old) (Figure 3B and Figure S4, lower left panel). Relative to moderate RSV, severe RSV was characterized by significantly lower levels of the Breg metacluster, a cell type involved in inflammation control and immunotolerance in both younger (< 6 months old) and all infants (< 1 year old) (Figure 3B and Figure S4, lower right panel). Like hospitalized infants with moderate disease, those with severe RSV were marked by a significant increase in granulocyte subsets linked to progenitor‐like and immature phenotypes relative to both mild and moderate RSV disease in both younger (< 6 months old) and all infants (< 1 year old) (Figure 3B and Figure S4, lower right panel). Based on the cluster profile of these metaclusters, immature (cluster 129) and progenitor‐like (clusters 113, 114) cell subsets have proliferative status, uncharacteristic of mature granulocytes and decreased levels of myeloid lineage markers such as CD11b, CD24, CD66b, CD11c, CD16 relative to mature subsets (Figure 2 and Figure S1B). In addition, these clusters show specific patterns of CD62L and CD16 protein expression which allows their identification within the granulocyte subset, as previously reported by Cortjens et al.^50^ and Pillay et al.^51^ (Figure S1B).

Together, the immature granulocyte subpopulations observed in all infants hospitalized with RSV, in combination with weakened humoral, CD4 and CD8 T cell and innate‐lymphocyte response in mechanically ventilated infants, indicates an activation of emergency myelopoiesis and impaired adaptive immune response in severe RSV. In addition, decreased Breg levels in severe RSV, relative to moderate may indicate the importance of immunotolerance in managing the infection.

### 3.5 Differential analysis of cell cluster frequencies relative to PBMC cell subset between different patient groups

Differential analysis of cell cluster frequencies between patient groups showed increased neutrophil abundance in infants with moderate and severe RSV. For a detailed view of cellular changes within the PBMC compartment (i.e., excluding effects of changes in granulocyte subsets on relative proportions of other cell subtypes within the singlet gate), we performed a differential analysis of PBMC cell subsets relative to the PBMC gate as depicted in Figure S1, between different patient groups (Figure 3C–F). Remarkably, a significant upregulation of the progenitor‐like neutrophil subset was observed in infants with severe relative to mild RSV (Figure 3F: cluster 114). While unexpected within the PBMC compartment, this cell cluster may represent the low‐density neutrophils that are a hallmark of emergency granulopoiesis. Additionally, the following observations were noted.

Hospitalized infants show unfavourable monocyte profiles marked by downregulation of HLA‐DR+ monocytes and the appearance of immune‐depressed, immature‐like HLA‐DR^–^ monocytes. The immune profiles of hospitalized infants (with or without mechanical ventilation) were characterized by specific monocyte cell populations displaying low expression levels of HLA‐DR and proliferative status based on elevated levels of Ki67, an immature‐like monocyte cell subset.^52^, ^53^ We observed an increase in these monocyte subsets in moderate RSV disease relative to healthy infants (Figure 3D: clusters 221, 222, 236 and 238) and in severe, relative to mild, RSV disease (Figure 3E: clusters 221, 222 and 236). In contrast, monocyte subsets characterized by high HLA‐DR expression showed downregulation in moderate RSV disease relative to healthy infants (Figure 3D: clusters 204 and 256) and in severe, relative to mild RSV disease (Figure 3E: cluster 204).

*Increase in inflammatory monocytes in moderate and severe RSV disease (hospitalised infants)*. Moderate RSV disease displayed a higher abundance of intermediate monocyte subset, characterized by high CD69, relative to healthy infants (Figure 3D: cluster 156), a subset associated with an inflammatory role,^54^ and CD69‐promoted tissue infiltration and retention.^55^, ^56^ Furthermore, we also observed higher levels of a cell subset representing CD141+ monocytes in severe, relative to mild, RSV disease (Figure 3E: cluster 223). The CD141+ monocytes have been associated with higher production of the inflammatory cytokines IL‐1β, IL‐6 and TNF and a reduction in FoxP3+ Treg skewing in vitro.^57^

*Immunomodulatory role of Treg and Breg in hospitalized infants*. Along with CD8 and CD4 Th1 subsets, we observed upregulation of effector Treg cells CD45RO+CD28+Ki67+ Treg in moderate RSV disease relative to healthy infants (Figure 3D: cluster 140), peaking in moderate RSV but declining in severe cases. Hospitalized infants also show high levels of Breg population relative to healthy infants (Figure 3D: cluster 84), while infants on mechanical ventilation had lower levels of CD123‐Breg cells relative to infants hospitalized without mechanical ventilation (Figure 3F: cluster 83). Together, the differential abundance of Treg and Breg cell subsets may indicate the importance of immunomodulation and suppression of inflammation in RSV severity.

*Moderate and severe RSV is characterized by a decrease in HLA‐DR+ B cells*. The B cell subsets characterized by high HLA‐DR expression (clusters 1, 50, 17, 82 and 49) progressively decreased in abundance in infants with a higher degree of disease severity: in mild RSV disease relative to healthy infants (Figure 3C: cluster 50), in moderate RSV disease relative to healthy infants (Figure 3D: clusters 1, 17, 49, 50 and 82), and severe relative to mild (Figure 3E: cluster 50) and relative to moderate RSV disease (Figure 3F: clusters 50 and 65). In parallel to the depletion of HLA‐DR+ B cells, hospitalized infants with moderate RSV showed an increase in B cell subsets characterized by low HLA‐DR expression relative to healthy infants (Figure 3D: clusters 69 and 100). As HLA‐DR is vital for B‐T cell interactions and antibody production, reduced HLA‐DR+ B cells in hospitalised infants might hinder the humoral response in severe RSV.

### 3.6 Random forest identifies HLA‐DR^Low^ monocytes as a hallmark of severe RSV disease

**FIGURE 4:**
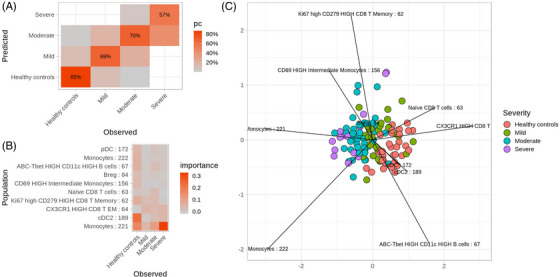
Random Forest analysis identifies HLA‐DR^Low^ monocytes (cluster 221) as the most important cell subset for classifying severe respiratory syncytial virus (RSV) disease in peripheral blood mononuclear cells (PBMCs). (A) Heatmap representing a confusion matrix, indicating the percentage of cases with correct random forest classifier's predictions and vice versa. In the confusion matrix, the columns represent the true patient group labels versus the predicted ones in the rows, with the diagonal representing the percentage of times when predictions match the true label. (B) The heatmap lists cell subsets (rows) most important for patient group classification (columns) and indicates the cell subset association with different RSV disease severity groups. (C) A compositional biplot provides a graphical representation of patient similarity across patient groups and of cell subsets driving their separation, the length of the line corresponding to the importance of the feature. Line labels correspond to cell clusters (name and cluster number included).

To classify different patient groups based on their immune profiles and identify cell subpopulations correlating with disease severity, we applied the supervised machine learning algorithm random forest,^58^ which builds multiple decision trees and takes their majority vote for classification and prediction (Figure 4). Using the cell cluster frequencies relative to the total PBMC subset as an input, the random forest algorithm was trained to classify infants in clinically annotated RSV disease severity groups, with the correct assignment to their respective group for 85% of healthy infants, 69% of infants with mild, 70% of infants with moderate and 57% of infants with severe RSV disease (overall error rate 28.8%, Figure 4A). Most infants that the random forest model incorrectly assigned into this latter group were infants with moderate RSV disease. The lowest predicting power of random forest was thus observed when distinguishing between moderate RSV disease versus severe RSV disease. HLA‐DR^Low^ monocytes (cluster 221) are the most important cell subset for the classification of severe RSV disease, whereas cDCs are the most important cell subset for the classification of healthy infants (Figure 4B). Based on patient grouping on the compositional biplot, we observed a gradual shift from healthy infants to infants with RSV disease with increasing RSV severity (Figure 4C). Healthy infants are grouped on the right side of the biplot driven by the vectors indicating the abundance of APC, pDC and cDC2 and ABC B cells and naïve CD8 T cells, and closely positioned to the non‐hospitalized RSV patient group, indicating immune profile similarity. In contrast, infants with severe RSV disease occupy the left side of the biplot driven by the vectors indicating the abundance of several monocyte subsets, including HLA‐DR^Low^ clusters (221 and 222).

Overall, with the exception of patients with severe RSV (57% prediction accuracy), the random forest was able to correctly predict clinical patient groups based on the profiled cell subsets, and specifically identified HLA‐DR^Low^ monocyte subsets as an important factor for characterizing severe RSV in infants (clusters 221 and 238).

The analysis was also replicated using the cell cluster frequencies relative to the total cell count (singlets gate) as an input with similar results for RSV disease severity based on ReSVinet classification (Figure S5A,B) and length of hospital stay (Figure S5C,D). The analyses reiterate the role of immature (cluster 129) and progenitor neutrophils (clusters 113 and 114) and HLA‐DR^Low^ monocytes (cluster 221) in the identification of severe RSV disease and indicate the predictive value of these cells for the length of stay in the hospital.

### 3.7 Functional marker analysis identifies increased p‐STAT3 activation in T cell, granulocyte and monocyte cell subsets

**FIGURE 5:**
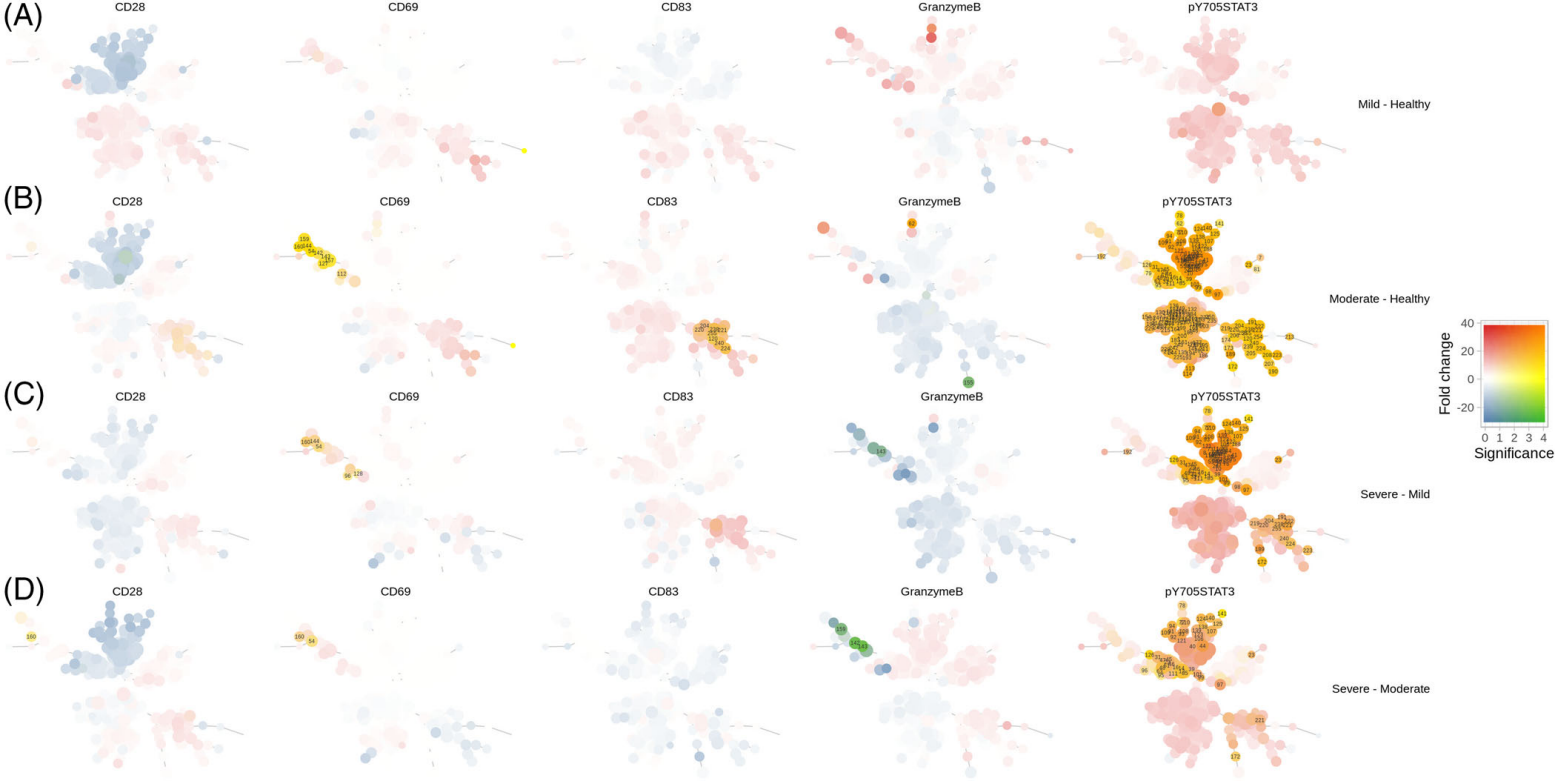
Differential analysis of functional marker expression levels reveals significant upregulation of *p*‐Y705‐STAT3 in T cell subsets, granulocytes and monocytes in severe respiratory syncytial virus (RSV) disease, among others. Results of differential analysis of cell cluster median marker expression between RSV severity groups are visualized as Tree Blend plots: for each cluster 2 color scales are built corresponding to estimated fold change (blue to red for depleted and enriched clusters, respectively) and significance (from white to yellow for the negative log_10_ of the false discovery rate [FDR]‐adjusted *p*‐value) and mixed to generate a set of colors representative of both variables. For each contrast, the clusters in the Minimum Spanning Tree projection are coloured after the mixed colours corresponding to the results of the significance test. (A) Mild disease versus healthy controls; (B) Moderate disease versus healthy controls; (C) Severe disease versus mild disease; (D) Severe disease versus Moderate disease. For the tree blend cell cluster legends, please refer to Figure 2A.

To assess the activation and functional status of immune cell subsets in severe RSV, we compared protein expression levels of several functional markers across the patients' groups (Figure 5). No significant difference was observed in mild RSV disease relative to healthy controls (Figure 5A). We observed upregulation of p‐STAT3 in numerous cell subsets in patients with moderate RSV relative to healthy infants (Figure 5B), with the highest upregulation observed in naïve CD4 T cell subset, Treg and CD279 positive CD4 T cells. Similarly, patients with severe RSV displayed significant upregulation of p‐STAT3 in CD4 and CD8 naïve T cells, Treg, CD279 high CD4 T cells, as well as in monocyte subsets relative to patients with mild and moderate RSV (Figure 5C,D). In addition, we observed differential regulation of granzyme B levels depending on the RSV disease severity. Patients with moderate RSV showed an increase in granzyme B in CD8 central memory (Ki67) (cluster 62) (Figure 5C), while patients with severe RSV showed downregulation of granzyme B in NK cell subsets relative to patients with moderate (Figure 5D, clusters 159, 142 and 147) and mild RSV (Figure 5C, cluster 142). Several monocyte subsets, including classic monocytes (clusters 204, 220, 255 and 120), progenitor‐like monocytes (clusters 221 and 222) and CD141 monocytes (clusters 224 and 240) showed significant upregulation of CD83 activation marker in patients with moderate RSV relative to healthy infants (Figure 5B). Significant upregulation of CD69 was observed in NK and TCRγδ cell subsets in patients with moderate RSV relative to healthy infants (Figure 5B: clusters 54, 127, 142, 142, 144, 157, 159, 160 and 112), and in severe RSV disease relative to mild disease (Figure 5C: clusters 54, 96, 128, 144 and 160) and relative to moderate RSV disease (Figure 5D: clusters 54 and 160) indicating activation of NK and TCRγδ cell subsets. High CD69 expression in the absence of granzyme B can indicate that an alternative method of cytotoxicity is active such as death receptor‐mediated targeted killing.^59^

### 3.8 Whole blood transcriptomics analysis identifies activation of myeloid cells in severe RSV disease

**FIGURE 6:**
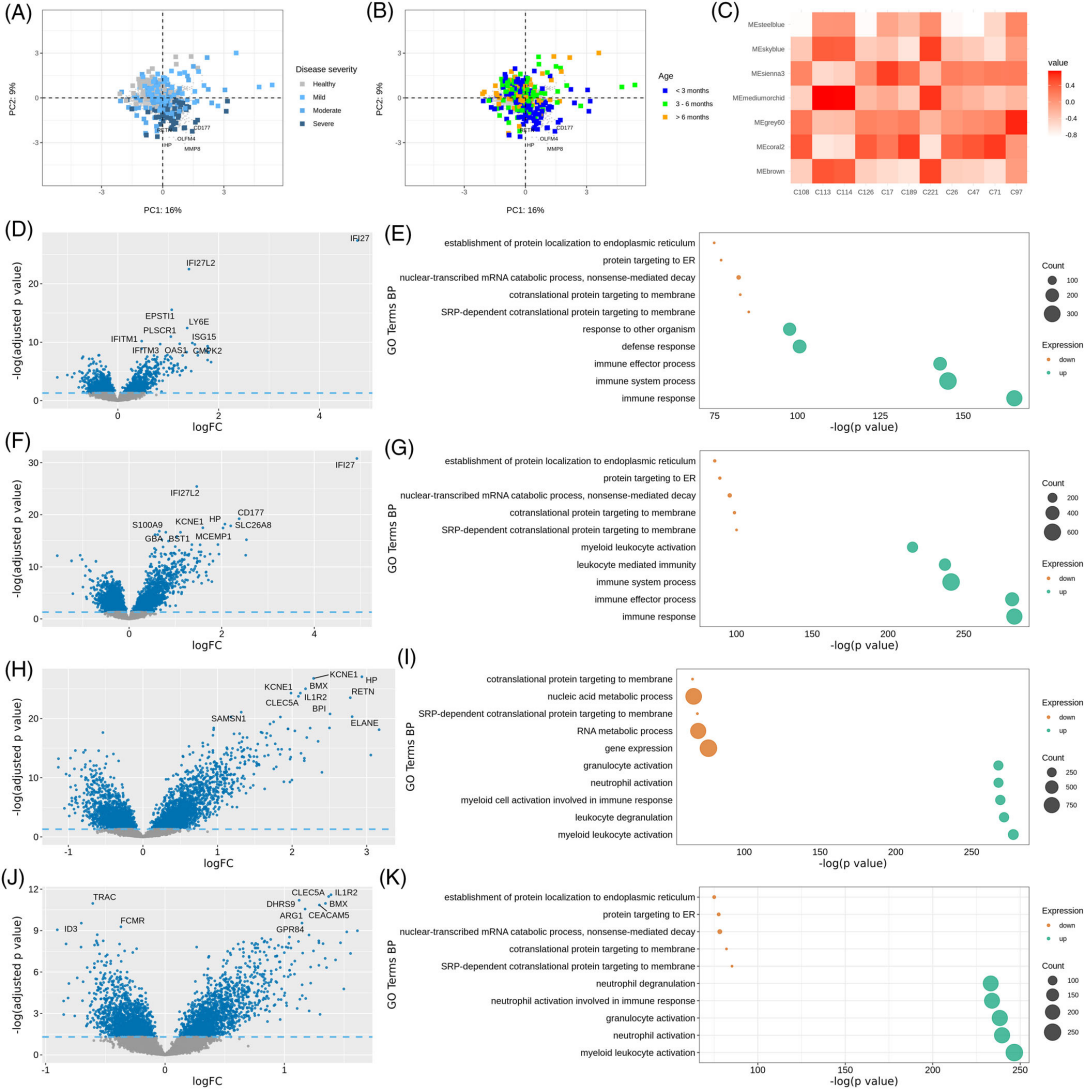
Whole blood transcriptomics analysis differentiates severe from mild respiratory syncytial virus (RSV) disease: severe RSV disease is marked with myeloid cell activation pathways and genes. (A and B) Principal component analysis (PCA) plot demonstrating the transcriptional similarity across the patient groups (unsupervised analysis). The PCA plot shows the first two principal components (PC1 and PC2) that explain 16% and 9% of the variation in the dataset, respectively. The top genes driving the variation in the transcriptomics profiles of the samples are labelled; samples are coloured by the infant disease severity class (A) and age groups (B). (C) Gene modules are shown on the y‐axis and cell subsets on the x‐axis. The highest correlation is seen with the module medium orchid and clusters 113 and 114 (progenitor neutrophils), and cluster 221 (HLA‐DR^Low^ and proliferative monocytes). Correlation values are shown from −1 (blue) to 1 (red). (D, F, H and J) Volcano plots (left side) derived from the supervised analysis showing the differential gene expression and bubble plots (E, G, I and K) derived from Gene ontology (GO) analysis showing the top 10 biological pathways for upregulated and downregulated genes of mild RSV disease compared with healthy controls (D and E), moderate RSV disease compared with healthy controls (F and G), severe RSV disease compared with mild RSV disease (H and I) and severe RSV disease compared with moderate RSV disease (J and K). The top 10 genes with the lowest false discovery rate (FDR)‐corrected *p*‐value are shown on the volcano plots.

We hypothesized that the observed emergency myelopoiesis in infants with severe RSV might be correlated with, and be reflected by, a set of gene expression markers in whole blood. Unsupervised PCA on whole blood transcriptomics data from infants at RSV visits and healthy controls (*N* = 268 samples, Table S2) revealed genes that drive the most pronounced variation in the gene expression dataset. Transcriptomics profiles from infants with severe RSV disease (*N* = 48) clearly differed from moderate (*N* = 92), mild (*N* = 72) and healthy infants (N = 56) (Figure 6A). The top five genes that drive this difference are *CD177* (CD177), *HP* (haptoglobin), *MMP8* (metallopeptidase 8), *OLFM4* (olfactomedin 4) and *RETN* (resistin) (Figure 6A). The effect of age was not the prominent driver of these genes (Figure 6B). Other potentially confounding factors, such as gender or site of sample collection, and timing of sampling, were not revealed as prominent drivers of variation in the transcriptomics dataset (data not shown).

A WGCNA using the overlapping subjects between single‐cell immune profiling and the transcriptomics data identified 66 gene modules comprising clusters of highly correlated genes. These modules were then used in a correlation analysis with measures of disease severity (ReSVinet severity score, hospitalization need, usage of mechanical ventilation) and risk factors for disease severity (age and sex) (Figure S6). Next, the modules were annotated with the cell clusters/bubbles analysis. The most significant gene modules which correlate (positively or negatively) with RSV disease severity and cell clusters are shown in Figure 6C. Modules named “sky blue”, “medium orchid” and “brown” were associated the most with progenitor cell clusters 113 (*r* = 0.75) and 114 (*r* = 0.73), which are assigned to OLFM4+ and OLFM4– progenitor neutrophils, respectively and cluster 221 (0.64), which is assigned to progenitor monocytes. These modules are also associated the most with severe RSV disease. The GO association of these modules were regulation of T cell differentiation in the thymus for the sky‐blue module, leukocyte cell activation involved in immune response for the brown module and neutrophil degranulation for the medium‐orchid module. Of note, these modules correlated negatively with conventional type 2 dendritic cells (cluster 189), classic B cells (cluster 17), naïve CD4 (cluster 71), Th17 cells (cluster 108), naïve CD8 T cells (cluster 47), naïve CD4 T cells (cluster 26) and CD161+ CD8 T cells (cluster 126; unassigned) (Figure 6C).

Age was positively associated with module “grey60”, which is associated with plasma cells and involves the genes in the adaptive immune response; and negatively associated with module “medium orchid” which is associated with neutrophil degranulation (Figure S6). This observation corresponds to the high innate immune response but lower adaptive immunity in younger infants. To explore further the effect of disease severity on the genes in module “medium orchid” at the young age group, we compared expression values in infants younger than 3 months old in the whole transcriptomics dataset (Table S2). Neutrophil‐associated genes (such as ARG1, BPI, OLFM4 and MMP8) showed high expression levels in severe RSV disease compared to milder forms and healthy controls and showed negligible difference between mild disease and healthy controls, at young age (Figure S7).

Supervised differential expression analysis (limma), comparing mild RSV disease with healthy controls, characterized upregulation of IFN response genes (e.g. *IFI27*, *IFI27L2, ISG15* and *IFI44L*) (Figure 6D) and of GO pathways related to antiviral immune defence, including immune and defence response (Figure 6E). Moderate RSV disease is characterized by activation of the IFN immune response combined with modulation of genes related to myeloid leukocyte activation (Figure 6G). Furthermore, supervised differential expression analysis unveiled an upregulation of genes including *BPI*, *HP* and *RETN* in severe RSV disease relative to mild RSV (Figure 6H), similar to the genes identified in the unsupervised analysis (Figure 6A). GO biological pathways (BP) analysis based on the differentially expressed genes in severe RSV disease relative to mild and moderate RSV highlighted an upregulation of the myeloid leukocyte activation and neutrophil activation pathways (Figure 6I,K).

Incorporating the PLS‐DA analysis into our methodology, we trained a sparse PLS‐DA model on our data using the disease severity groups as the dependent variable. We discerned a distinct separation between the groups, illustrated in (Figure S8). Intriguingly, our analysis identified IFI27 and IFI27L2 as pivotal genes that show high expression in all samples and groups except for healthy controls, with relatively low expression in severe disease when compared to mild and moderate disease. Similarly, neutrophil activity‐related genes such as MMP8 drive mapping along component one, indicating higher expression of these genes in patients with moderate and severe disease when compared to mild disease and healthy controls. These genes manifested prominently in the mild and moderate RSV disease groups, showcasing a robust antiviral response. In contrast, their expression was comparatively diminished in the control and severe RSV disease groups. This trend reinforces our immune profiling results, emphasizing a heightened antiviral defence in mild and moderate instances of RSV disease and a lacklustre defence in severe cases.

Thus, whole blood transcriptomic analysis indicates the activation of a robust antiviral response in mild RSV disease, whereas progression to severe disease activates the myeloid compartment of the immune system, in accordance with the whole blood single‐cell analysis.

## 4 DISCUSSION

In our study, we explored immune responses in infants with mild (outpatients), moderate (inpatients without mechanical ventilation) and severe (inpatients with mechanical ventilation) RSV disease to identify potential biomarkers linked to disease severity. We found that infants with mild RSV symptoms exhibit an immune response marked by cytotoxic CD8 T cells and adaptive immunity activation. All hospitalized infants (moderate and severe RSV disease) show reduced antiviral responses and increase of inflammatory monocytes and HLA‐DR^Low^ B cells, and signs of neutrophil activation. Hospitalised infants with mechanical ventilation (severe RSV disease) display a high neutrophil/lymphocyte ratio (or lymphopenia), the proliferation of Ki67+ HLA‐DR^Low^ monocytes, progenitor and immature neutrophils, and a diminished T cell response, pointing towards the activation of emergency myelopoiesis.

The use of the cell cycle marker Ki67+ in the study panel allowed us to explore the proliferative status of monocytes and neutrophils, among others. The proliferative status of monocytes and neutrophils in peripheral circulation is associated with an immature state and indicates immature cell recruitment to the peripheral circulation, a hallmark of emergency myelopoiesis. Such dysregulated myeloid profile may contribute to the insufficient antiviral immune response; however, the severe disease phenotype can also be the result of and hyper‐inflammatory environment.^60^

Proliferative (Ki67^High^) blood monocytes have also been reported in coronavirus disease 2019 (COVID‐19)‐infected patients,^61^, ^62^ suggesting that the observed immune profile is not specific or solely limited to severe RSV disease. In their longitudinal analysis, Mann et al.^61^ identified increased Ki67 expression in blood monocytes, reduced COX‐2 expression and a high neutrophil to T cell ratio as early predictors of disease severity.^61^ In particular, the authors showed the presence of activated monocytes early upon hospital admission which gradually reduced as patients progressed towards more severe disease, indicating the potential use of monocytes as a predictive tool to stratify and select patients that will likely benefit most from a therapeutic intervention.^61^ Additional longitudinal studies and investigation of immune profiles of infants with severe RSV disease will be necessary to appreciate whether dysregulated monocytes can be used as early predictors of severe RSV disease.

One of the mechanisms proposed to explain the lymphopenia in severe virus infections is the overproduction of IL‐6 driving the activation of the STAT3 pathway, leading to impaired lymphopoiesis by directly affecting hematopoietic stem cells.^63^ Conversely, IL‐6 release invokes neutrophil mobilization from bone marrow,^64^ for a hallmark of emergency granulopoiesis.^65^, ^66^ In this study, we observed significant upregulation of p‐Y705‐STAT3, a downstream target of the IL‐6R pathway, in numerous cell subsets including T cell subsets, granulocytes and monocytes, while the most pronounced effect was in naïve CD4 and naïve CD8 T cells. In contrast, no significant upregulation of p‐Y705‐STAT3 was observed in non‐hospitalized RSV‐infected infants. Interestingly, IL‐6 signalling has also been associated with downregulation of HLA‐DR expression in monocytes and B cells in COVID‐19.^60^, ^67^

Various neutrophil subsets have been associated with severe RSV disease, including mature, immature, progenitor and suppressive subsets, which highlight different roles of neutrophils depending on disease severity level.^50^ The progenitor neutrophil subset was characterized by high Ki67 expression, as well as by low expression of granulocyte lineage markers such as CD24, CD11b, CD66b and CD11c. The premature release of immature myeloid cells from the bone marrow is thought to be associated with innate immune dysfunction (or vice versa), alongside more extensive lung damage and poorer clinical outcomes.^61^ Integrated analysis of single‐cell immune profiling and whole blood transcriptomics demonstrated that myeloid/neutrophil activation in the blood of infants with severe RSV disease is strongly associated with highly increased expression of a distinct set of genes (especially *ARG1, AZU1, BPI, CEACAM8, DEFA1, DEFA3, ELANE, LTF, MMP8, OLFM4* and *TCN1*), demonstrating the potential of transcriptomics as a biomarker for discerning RSV disease severity.

Furthermore, here we report the emergence of HLA‐DR^Low^ B cells in severe RSV disease. HLA‐DR^Low^ B cells and monocytes have been associated with immunodepression or immunoparalysis, and have been previously reported in sepsis^68^ and COVID‐19.^67^ In the latter case, the downregulation of HLA‐DR has been associated with STAT3 mediated effect of IL‐6.^67^ HLA‐DR is essential for B cell interaction with T cells, subsequent B cell activation and the generation of antibody‐producing cells. Hence, it can be assumed that a decreased proportion of HLA‐DR+ B cells and the appearance of HLA‐DR‐ B cells in moderate and severe RSV disease may significantly affect the humoral response in severe RSV in infants.

The observation that severe RSV is often seen in very young infants (< 3 months old) may signify an underlying immature immunity to infections, including an imbalance of Th1/Th2 immunity and low levels of antibodies, all leading to an immune environment that makes the infant more susceptible for severe infections. Likewise, in this study, the infants using mechanical ventilation were significantly younger than the non‐hospitalized and non‐ventilated infants. To tackle this possible bias, we conducted complementary analyses in infants younger than 6 months old and infant age was included as a covariate in our statistical analyses.

It is unknown whether the observed immature monocytes or neutrophils are predictive of infants with a high risk for severe RSV disease or rather a consequence and hence a correlate of severe disease. While our study describes in detail the immune environment and reveals correlated markers of severe versus mild RSV disease and healthy infants, its case‐control design does not allow us to speculate on potentially predictive biomarkers of severe RSV disease. The differences in immune profiles could also be attributed to the time of sampling at the disease presentation. Although we see a bigger variability in the time of sampling in severe RSV disease, the time of sampling is comparable between the disease groups. To enable us to use biomarkers as a predictor of disease, prospective analysis of disease severity (mild cases evolving into severe cases of disease) and longitudinal sampling would be necessary.

Whole blood analysis provides a reflection of the systemic response to the viral infection but is only secondary to the initial host immune response that is elicited in the lung mucosa where RSV infection occurs. Therefore, the involved cell subsets and associated transcriptomic markers could be different in the lung versus our findings in blood. Immune profiling of mucosal tissue and comparison with the results found in whole blood was not possible in our study but is a potential avenue for future studies.

Our study has limitations. Firstly, It is known that the use of mechanical ventilation enhances lung inflammation, and therefore alters immune response.^69^, ^70^ In this study, we classified severe RSV disease by hospitalization and the use of mechanical ventilation; therefore, the elevated immune response in severe RSV disease might be influenced by mechanical ventilation. Secondly, bacterial co‐infections or the presence of other respiratory viruses might influence RSV disease presentation. A comprehensive analysis of a panel of respiratory viral and bacterial infections was, unfortunately, not covered in our study. Thirdly, considering that we only recruited infants under 1 year of age, the likelihood of prior RSV infection in these infants is minimal, however, prior RSV infections in some of the older children are conceivable. Finally, we acknowledge that our study's conclusions are based on phenotypic observations in whole blood. While emphasizing the significance of phenotypic assays As the phenotypic patterns offer valuable information, complementary functional assays will be required to gain further insights into the appearance of emergency myelopoiesis in severe RSV disease.

To our knowledge, this is the first study to provide phenotypic evidence in whole blood for the involvement of emergency myelopoiesis in severe RSV disease. Single‐cell immune profiling and integrated transcriptomic analyses revealed specific cell subsets and genes that correlate with mild or severe RSV disease, warranting validation in independent cohorts.

## AUTHOR CONTRIBUTIONS

N.Z., D.Ö., Y.A., J.MG. and J.A. designed the study, collected data and were involved in the substantial interpretation and preparation of the reports of the analyses and manuscript draft. S.B.D., J.G.W., R.S.T., H.R., L.B., P.J.M.O., F.M.‐T., A.J.P. and J.A. designed, conducted, and supervised the clinical studies. N.Z., D.Ö., K.T. and G.V. designed the CyTOF antibody panel and were involved in data analysis and interpretation. N.Z. performed the CyTOF analysis, manual cell gating and provided input for computational CyTOF data analysis. Y.A. performed the computational analyses of the CyTOF data. D.Ö. and J.MG. performed the transcriptomic analyses. M.C. advised on the biostatistical analyses. All authors were involved in the analysis plan, critically reviewed the manuscript and contributed to and approved the final version.

## RESCEU INVESTIGATORS

Harish Nair, Harry Campbell (University of Edinburgh), Philippe Beutels (Universiteit Antwerpen), Louis Bont (University Medical Center Utrecht), Andrew Pollard (University of Oxford), Ryan Thwaites, Peter Openshaw (Imperial College London), Federico Martinon‐Torres, Carmen Rodriguez‐Tenreiro Sánchez, Irene Rivero‐Calle, Ana Dacosta Urbieta, Fernando Caamaňo, Sara Pischedda (Servicio Galego de Saude), Terho Heikkinen (University of Turku and Turku University Hospital), Adam Meijer (National Institute for Public Health and the Environment), Thea K Fischer (Statens Serum Institut), Maarten van den Berge (University of Groningen), Carlo Giaquinto (PENTA Foundation), Michael Abram (AstraZeneca), Kena Swanson (Pfizer), Bishoy Rizkalla (GlaxoSmithKline), Charlotte Vernhes, Scott Gallichan (Sanofi), Jeroen Aerssens (Janssen), Veena Kumar (Novavax), Eva Molero (Team‐It Research).

## CONFLICT OF INTEREST STATEMENT

D.Ö., N.Z., K.T., M.C., G.V., Y.A. and J.A. are employees of Janssen Pharmaceutica NV.

S.B.D. has been an investigator for clinical trials of vaccines and antimicrobials for pharmaceutical companies including AstraZeneca, Merck, Pfizer, Valneva, Iliad, Sanofi and Janssen. All funds have been paid to his institution. He has previously sat on RSV advisory boards for Sanofi and Merck.

L.B. has regular interaction with pharmaceutical and other industrial partners. He has not received personal fees or other personal benefits. UMCU has received major funding (> €100,000 per industrial partner) for investigator‐initiated studies from AbbVie, MedImmune, AstraZeneca, Sanofi, Janssen, Pfizer, MSD and MeMed Diagnostics. UMCU has received major funding for the RSV GOLD study from the Bill and Melinda Gates Foundation. UMCU has received major funding as part of the public‐private partnership IMI‐funded RESCEU and PROMISE projects with partners GSK, Novavax, Janssen, AstraZeneca, Pfizer and Sanofi. UMCU has received major funding from Julius Clinical for participating in clinical studies sponsored by MedImmune and Pfizer. UMCU received minor funding (€1,000‐25,000 per industrial partner) for consultation and invited lectures by AbbVie, MedImmune, Ablynx, Bavaria Nordic, MabXience, GSK, Novavax, Pfizer, Moderna, AstraZeneca, MSD, Sanofi, Genzyme, Janssen. L.B. is the founding chairperson of the ReSViNET Foundation.

P.J.M.O. received honoraria GSK, Pfizer Inc, Sanofi Pasteur, Seqirus, Moderna and Janssen for participation in advisory boards and expert meetings and for acting as a speaker in congresses outside the scope of the submitted work. P.J.M.O. is also a principal investigator in the INFLAMMAGE trial co‐funded by the Medical Research Council UK and GSK as part of the EMINENT consortium to promote inflammation research.

F.M.‐T. received honoraria from the GSK group of companies, Biofabri, Pfizer Inc, Sanofi Pasteur, MSD, Seqirus and Janssen for taking part in advisory boards and expert meetings and for acting as a speaker in congresses outside the scope of the submitted work. F.M.‐T. has also acted as principal investigator in randomized controlled trials of the above‐mentioned companies as well as Ablynx, AstraZeneca, Gilead, Regeneron, Roche, Abbott, Novavax and MedImmune, with honoraria paid to his institution.

A.J.P. is chair of the UK Department of Health and Social Care's (DHSC) Joint Committee on Vaccination and Immunisation (JCVI), NIHR Senior Investigator, member of the Academy of Medical Sciences (AMS) and was a member of WHO's SAGE until January 2022. A.J.P.'s institution received funding from the European Commission's IMI programme for the conduct of this study. Oxford University has entered a partnership with AstraZeneca for the development of COVID‐19 vaccines. The views expressed in this article do not necessarily represent the views of DHSC, JCVI, NIHR, AMS or WHO.

## Supporting information

Supporting Information

Supporting Information

Supporting Information

Supporting Information

Supporting Information

Supporting Information

Supporting Information

Supporting Information

Supporting Information

## Data Availability

CyTOF fcs files are being deposited in https://flowrepository.org (placeholder: https://flowrepository.org/id/FR‐FCM‐Z63W). Transcriptomics microarray dataset deposited https://www.ncbi.nlm.nih.gov/geo/ with the GSE246622 accession number. The original R codes are being deposited in https://github.com/JanssenDiscoveryOmics/RESCEU_ImmuneProfiling.
