# Supplementary material for: Single‐cell immune profiling reveals markers of emergency myelopoiesis that distinguish severe from mild respiratory syncytial virus disease in infants
Source: Clin Transl Med. 2023 Dec 19;13(12):e1507. doi: 10.1002/ctm2.1507 (PMC10731116; doi:10.1002/ctm2.1507)
Supplement: Supplementary file 1 — Supporting Information [file CTM2-13-e1507-s001.docx]

**SUPPLEMENTARY TEXT**

**Single-cell immune profiling reveals markers of emergency myelopoiesis that distinguish severe from mild respiratory syncytial virus (RSV) disease in infants**

Nevena Zivanovic^1, a^, Deniz Öner^1, a^, Yann Abraham^1, a^, Joseph McGinley^2, a^, Simon B. Drysdale^3^, Joanne G. Wildenbeest^4^, Marjolein Crabbe^1^, Greet Vanhoof^1^, Kim Thys^1^, Ryan S. Thwaites^5^, Hannah Robinson^2^, Louis Bont^4^, Peter J.M. Openshaw^5^, Federico Martinón-Torres^6,7,8^, RESCEU Investigators, Andrew J. Pollard^2, b^, Jeroen Aerssens^1, b^.

^1^Janssen Research & Development, Beerse, Belgium

^2^Oxford Vaccine Group, Department of Paediatrics, University of Oxford, and the NIHR Oxford Biomedical Research Centre, Oxford, United Kingdom

^3^Centre for Neonatal and Paediatric Infection, Institute for Infection and Immunity, St George’s, University of London, London, United Kingdom

^4^Department of Pediatric Infectious Diseases and Immunology, Wilhelmina Children’s Hospital, University Medical Center Utrecht, Utrecht, the Netherlands

^5^National Heart and Lung Institute, Imperial College London, London, United Kingdom

^6^Translational Pediatrics and Infectious Diseases, Pediatrics Department, Hospital Clínico Universitario de Santiago de Compostela, Santiago de Compostela, Spain

^7^Genetics, Vaccines and Infections Research Group (GENVIP), Instituto de Investigación Sanitaria de Santiago, University of Santiago de Compostela, Spain

^8^Centro de Investigación Biomédica en Red de Enfermedades Respiratorias (CIBERES), Instituto de Salud Carlos III, Madrid, España

^a^These authors contributed equally

^b^Correspondence: jaerssen@its.jnj.com

**ACKNOWLEDGEMENTS**

This work was supported by the Respiratory Syncytial Virus Consortium in Europe (RESCEU) project. RESCEU has received funding from the Innovative Medicines Initiative 2 Joint Undertaking (grant number 116019). This Joint Undertaking receives support from the European Union Horizon 2020 Research and Innovation.

Graphical abstract and Figure 1 were created with BioRender.com.

# Supplemental figure titles and legends

**Figure S1: Gating strategy of the single-cell proteomics analysis.**

(**A**) Gating strategy applied to separate cells into discrete populations based on shared marker expression, in the hierarchical order. Cell populations identified by this gating strategy are highlighted in biaxial contour plots. (**B**) Granulocyte subpopulation gating strategy using combinatorial expression of CD16 and CD62L. Single CD16 vs CD62L biaxial plot is colored with median marker expression of different myeloid lineage markers (CD11c, CD11b, CD66b and CD24), and Ki67 to visualize granulocyte subset myeloid marker expression levels.

**Figure S2:** **Confirmation of FlowSOM clusters based on the manually gated cell populations.**

Heatmap indicates the number of cells from manually gated cell populations present within each FlowSOM cluster normalized to the maximum number of cells in the cluster. This enables identification of clusters containing mixture if cell subtypes as identified by manual gating.

**Figure S3: Phenotyping of the metaclusters.**

(**A**) Heatmap representing the number of cells from each metacluster normalized to the total population size, enabling quick identification of bubbles that correspond to the majority of a given population. Y-axis shows the list of metaclusters, and the x-axis shows the list of manually gated cell populations. (**B**) Heatmap representing metacluster (y-axis) median marker expression scaled per marker (x-axis).

**Figure S4: Differential analysis of cell subsets for various comparisons of infants younger than 6 months old with different degrees of RSV disease severity**

Volcano plots show differential analysis of cell subsets for various comparisons of infants with different degrees of RSV disease severity. The estimated fold change is shown on x-axis and -log10 of adjusted p value is shown on y-axis. The horizontal lines represent FDR-adjusted p value < 0.05.

**Figure S5: Random Forest analysis to predict cell subsets that differentiates severe RSV disease in whole blood**.

(**A, C**) Heatmap representing a confusion matrix, indicating the percentage of cases with correct random forest classifier's predictions and *vice versa*. In confusion matrix the columns represent the true patients’ groups labels versus the predicted ones in the rows, with the diagonal representing the percentage of times when predictions match the true label for (**A**) different RSV disease severity groups based of the ReSVinet score: healthy, mild (< 7 points), moderate (>= 7 points and < 14 points), and severe (>= 14 points) and (**C**) the length of hospital stays, classified as short (<= 5 days) or long (> 5 days). (**B, D**) Compositional biplot provides graphical representation of patient similarity across patient groups and of cell subsets driving their separation for (**B**) different RSV disease severity groups based of the ReSVinet score: healthy, mild (< 7 points), moderate (>= 7 points and < 14 points), and severe (>= 14 points) and (**D**) the length of hospital stays, classified as short (<= 5 days) or long (> 5 days). The length of the line corresponding to the importance of the feature. Line labels correspond to cell clusters (name and cluster number included).

**Figure S6: Modules showing greater correlation with the disease severity.**

Heatmap showing the correlation of disease severity and gene modules. Clinical characteristics such as sex and age category (younger than 3 months old, between 3 and 6 months old, and between 6 and 12 months old), and disease severity classifications such as ReSVinet score, hospitalisation, and use of mechanical ventilation are shown on the x-axis. Gene modules identified by the Weighted Gene Correlation Network (WGCNA) are shown on the y-axis.

**Figure S7: Comparison of the expression levels of the selected genes in the “medium orchid” module between disease severity groups.**

Infants younger than 3 months of age were displayed in the graphs. Infants were grouped based on the hospitalization status as disease severity: Healthy controls, mild, moderate, and severe, with the colors shown in the legend. Expression values of the genes (A-H) *ARG1, AZU1, BPI, CEACAM8, ELANE, LCN2, MMP8, BPI*, were shown on the y-axis as Log_2_ intensity. One-way Anova (analysis of variance) analysis with TukeyHSD (honestly significant difference) multiple comparison of means (95 % confidence level) was applied to find the significant comparisons. Statistical significance is denoted as follows: * for p < 0.05, ** for p < 0.01, *** for p < 0.001, and 'n.s.' indicates not significant.

**Figure S8: PLS-DA shows separation of RSV disease groups.**

(A) Scatter plot showing the mapping of samples according to the sparse PLS-DA model into dimensions 1 and 2 using 100 most informative genes according to the sparse PLS-DA algorithm, colored according to RSV disease status. (B) Scatter plot of the feature importance of the 100 genes making in the sparse PLS-DA model, indicating the extent to which each gene drives projection onto component 1 and component 2.

**Table S1: Key resources table**

| **REAGENT or RESOURCE** | **SOURCE** | **IDENTIFIER** |
| --- | --- | --- |
| **Antibodies for CyTOF** |  |  |
| HI30 (CD45) | Fluidigm (Standard Bio) | *Cat#: 3089003B* |
| RPA-T4 (CD4) | BD Biosciences | *Cat#: 555344, RRID: AB_395749* |
| WM53 (CD33) | BD Biosciences | *Cat#: 555449, RRID: AB_395842* |
| HB15e (CD83) | BD Biosciences | *Cat#: 556854, RRID: AB_396525* |
| poly (IFI27) | Thermo Fisher | *Cat#: PA5-55997, RRID: AB_2642635* |
| UCHT2 (CD5) | Fluidigm (Standard Bio) | *Cat#: 3143007B* |
| ICRF44 (CD11b) | Fluidigm (Standard Bio) | *Cat#: 3144001B* |
| FN50 (CD69) | BD Biosciences | *Cat#: 555529, RRID: AB_395914* |
| J252D4 (CXCR5) | Biolegend | *Cat#: 356902, RRID: AB_2561811* |
| HIB-19 (CD19) | BD Biosciences | *Cat#: 555410, RRID: AB_395810* |
| 5A6.E9 (TCRγδ) | Thermo Fisher | *Cat#: TCR1061, RRID: AB_223500* |
| UCHL1 (CD45RO) | Fluidigm (Standard Bio) | *Cat#: 3149001B* |
| D4E3M (ARG1) | Cell Signaling Technology | *Cat#: 93668, RRID:AB_2800207* |
| 6H6 (CD123) | Fluidigm (Standard Bio) | *Cat#: 3151001B* |
| ML5 (CD24) | BD Biosciences | *Cat#: 555426, RRID: AB_395820* |
| DREG-56 (CD62L) | Fluidigm (Standard Bio) | *Cat#: 3153004B* |
| CD28.2 (CD28) | ML - BD Biosciences | *Cat#: 555725, RRID: AB_396068* |
| L161 (CD1c) | Biolegend | *Cat#: 331502, RRID: AB_1088995* |
| HCD14 (CD14) | Fluidigm (Standard Bio) | *Cat#: 3156019B* |
| 4/P-Stat3 (pY705-STAT3) | Fluidigm (Standard Bio) | *Cat#: 3158005A* |
| Bu15 (CD11c) | Fluidigm (Standard Bio) | *Cat#: 3159001B* |
| 4B10 (t-Bet) | Fluidigm (Standard Bio) | *Cat#: 3160010B* |
| B56 (Ki-67) | Fluidigm (Standard Bio) | *Cat#: 3161007B* |
| PCH101 (FOXP3) | Fluidigm (Standard Bio) | *Cat#: 3162011A* |
| D1E4M (OLMF4) | Cell Signaling Technology | *Cat#: 14369, RRID: AB_2798465* |
| HP3G10 (CD161) | Biolegend | *Cat#: 339902, RRID: RRID: AB_1501090* |
| A019D5 (CD127) | Fluidigm (Standard Bio) | *Cat#: 3165008B* |
| HI100 (CD45RA) | Fluidigm (Standard Bio) | *Cat#: 3166028D* |
| HIT2 (CD38) | Fluidigm (Standard Bio) | *Cat#: 3167001B* |
| L50-823 (GATA3) | BD Biosciences | *Cat#: 558686, RRID: AB_2108590* |
| 2A3 (CD25) | Fluidigm (Standard Bio) | *Cat#: 3169003B* |
| EH12.1 (CD279 (PD1)) | BD Biosciences | *Cat#: 562138, RRID: AB_10897007* |
| GB11 (Granzyme B) | Fluidigm (Standard Bio) | *Cat#: 3171002B* |
| 2A9-1 (CX3CR1) | Fluidigm (Standard Bio) | *Cat#: 3172017B* |
| 1A4 (CD141) | Fluidigm (Standard Bio) | *Cat#: 3173002B* |
| L243 (HLA-DR) | Fluidigm (Standard Bio) | *Cat#: 3174001B* |
| 29E.2A3 (CD274 (PD-L1)) | Fluidigm (Standard Bio) | *Cat#: 3175017B* |
| R19-760 (CD56) | Fluidigm (Standard Bio) | *Cat#: 3176013B* |
| RPA-T8 (CD8) | BD Biosciences | *Cat#: 555364, RRID: AB_395767* |
| UCHT1 (CD3) | BD Biosciences | *Cat#: 555330, RRID: AB_395737* |
| 80H3 (CD66b) | Abnova | *Cat#: MAB4157, RRID: AB_10632094* |
| 3G8 (CD16) | Fluidigm (Standard Bio) | *Cat#: 3209002B* |
| **Biological samples** |  |  |
| Whole blood from healthy adults (reference sample) | Janssen Biobank | N/A |
| Whole blood from RSV-infected infants | RESCEU infant birth cohort^28^, RESCEU infant case-control cohort^27^ | N/A |
| Whole blood from healthy infants | RESCEU infant case-control cohort^27^ | N/A |
| **Chemicals, peptides, and recombinant proteins** |  |  |
| Smart tube | Smart Tube, Inc | Cat#: 501351690 |
| Thaw-Lyse Buffer | Smart Tube, Inc | Cat#: 501351696 |
| UltraPure™ DNase/RNase-Free Distilled Water | Invitrogen™ | Cat#: 10977035 |
| PBS | Sigma-Aldrich | Cat#: 806552 |
| Lyse 2Buffer | Smart tube Inc | Cat#: 501351694 |
| Stain buffer | BD Biosciences | Cat#: 554657 |
| Maxpar 10X Barcode Perm Buffer | Standard Bio Tools | Cat#: 201057 |
| Cell-ID™ 20-Plex Pd Barcoding Kit | Standard Bio Tools | Cat#: 201060 |
| TruStain FcXTM | Biolegend | Cat#: 422302 |
| Maxpar® Cell Staining Buffer | Standard Bio Tools | Cat#: 201068 |
| BD Phosflow™ Perm Buffer III | BD Biosciences | Cat#: 558050 |
| Cell Acquisition Solution | Standard Bio Tools | Cat#: 201244 |
| EQ Four Element Calibration Beads | Standard Bio Tools | Cat#: 201078 |
| **Critical commercial assays** |  |  |
| Maxpar® Antibody Labeling Kit | Standard BioTools | Cat#: 201300 |
| QIAsymphony PAXgene Blood RNA Kit | QIAGEN | Cat#: 762635 |
| Clariom™ GOScreen microarray | Thermo Fisher | Cat#: 952361 |
| Thermo Fisher; GeneChip Pico Reagent Kit | Thermo Fisher | Cat#: 902622 |

**Table S2:** **Clinical and demographic characteristics of the infants for the transcriptomics analyses**

|  | Healthy controls | MILD  (RSV-infected, non-hospitalized) | MODERATE  (RSV-infected, hospitalized without mechanical ventilation) | SEVERE  (RSV-infected, hospitalized with mechanical ventilation) |
| --- | --- | --- | --- | --- |
| *Infants* | *(N=56)* | *(N=72)* | *(N=92)* | *(N=48)* |
| **Age at enrolment** |  |  |  |  |
| Below 3 months | 8 (14.3%) | 13 (18.1%) | 46 (50.0%) | 43 (89.6%) |
| 3 to < 6 months | 18 (32.1%) | 16 (22.2%) | 25 (27.2%) | 3 (6.3%) |
| 6 to 12 months | 30 (53.6%) | 43 (59.7%) | 21 (22.8%) | 2 (4.2%) |
| **Sex** |  |  |  |  |
| Female | 18 (32.1%) | 36 (50%) | 40 (43.5%) | 18 (37.5%) |
| Male | 38 (67.9%) | 36 (50%) | 52 (56.5%) | 30 (62.5%) |
| **Site** |  |  |  |  |
| IMPERIAL | 4 (7.1%) | 2 (2.8%) | 0 (0%) | 3 (6.3%) |
| OXFORD | 25 (44.6%) | 23 (31.9%) | 57 (62.0%) | 3 (6.3%) |
| UMCU | 27 (48.2%) | 39 (54.2%) | 25 (27.2%) | 38 (79.2%) |
| SERGAS | 0 (0%) | 8 (11.1%) | 10 (10.9%) | 4 (8.3%) |
| **ReSVinet score** |  |  |  |  |
| Mean (SD) | NA | 3.73 (2) | 8.93 (2.96) | 13.9 (3.25) |
| Median [Min, Max] | NA | 3.5 [0, 10] | 9.00 [2, 15] | 15 [6, 18] |
| Missing | NA | 6 (8.3%) | 0 (0%) | 0 (0%) |
| **Respiratory support** |  |  |  |  |
| No | NA | 50 (69.4%) | 20 (21.7%) | 0 (0%) |
| Yes | NA | 0 (0%) | 72 (78.3%) | 48 (100%) |
| Missing | NA | 22 (30.6%) | 0 (0%) | 0 (0%) |
| **Timing of sampling** |  |  |  |  |
| Mean (SD) | NA | 3.25 (1.32) | 4.12 (1.55) | 4.31 (2.51) |
| Median [Min, Max] | NA | 3.00 [0, 7] | 4.00 [1, 8] | 4.00 [0, 16] |
| Missing | NA | 4 (5.6%) | 1 (1.1%) | 0 (0%) |

Data are represented as number of subjects (%) unless otherwise is stated.
