# Supplementary figures and images for: Single‐cell immune profiling reveals markers of emergency myelopoiesis that distinguish severe from mild respiratory syncytial virus disease in infants
Source: Clin Transl Med. 2023 Dec 19;13(12):e1507. doi: 10.1002/ctm2.1507 (PMC10731116; doi:10.1002/ctm2.1507)

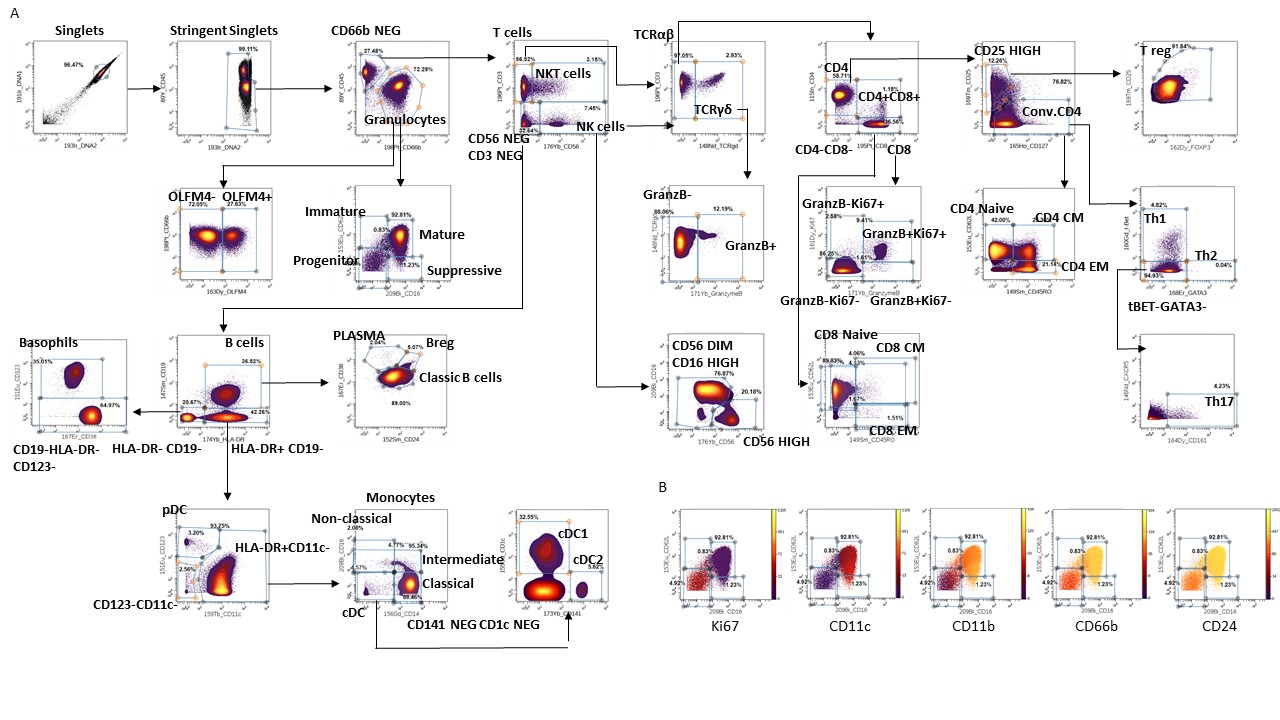

Supplement: Supplementary file 2 — Supporting Information [file CTM2-13-e1507-s006.jpeg]

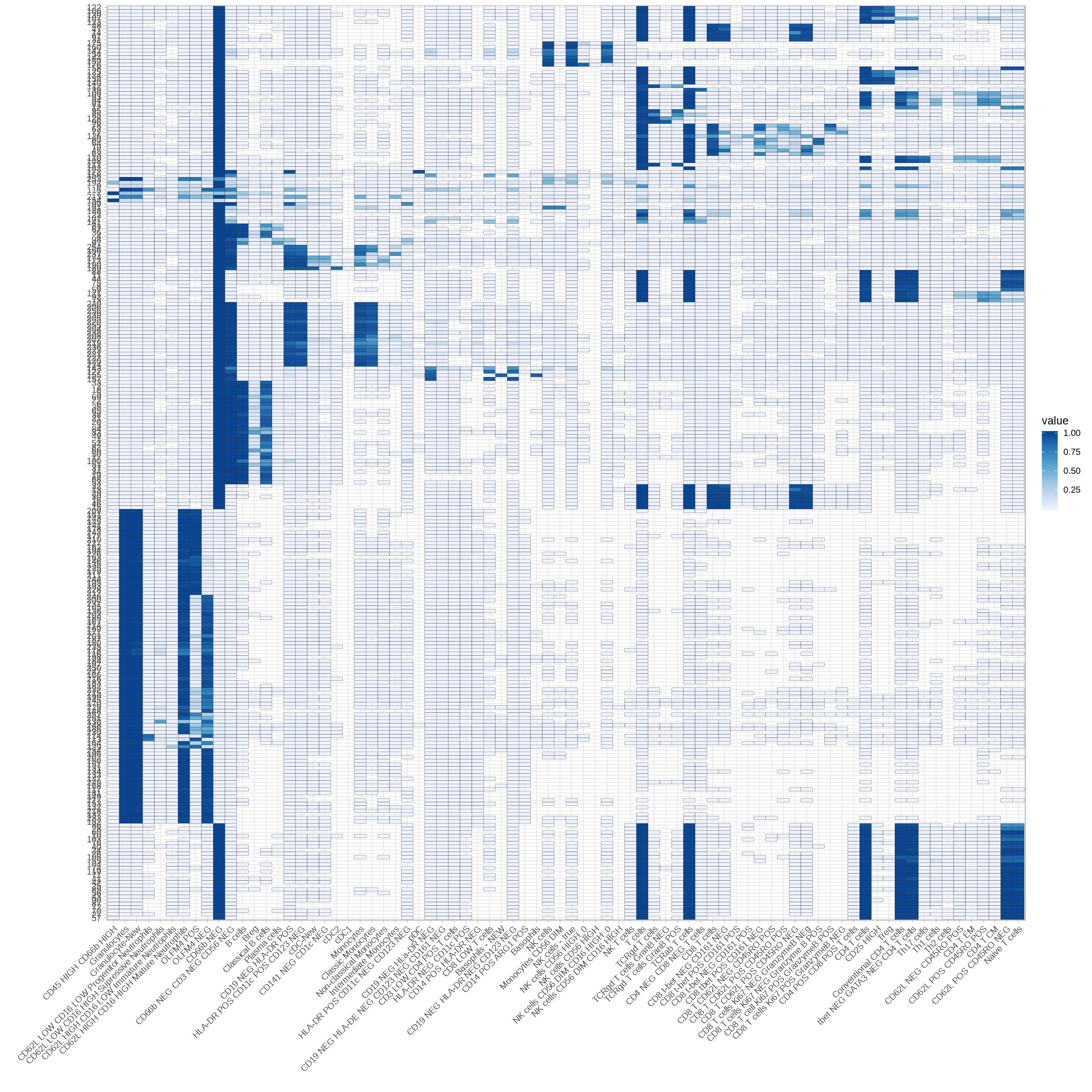

Supplement: Supplementary file 3 — Supporting Information [file CTM2-13-e1507-s002.tif]

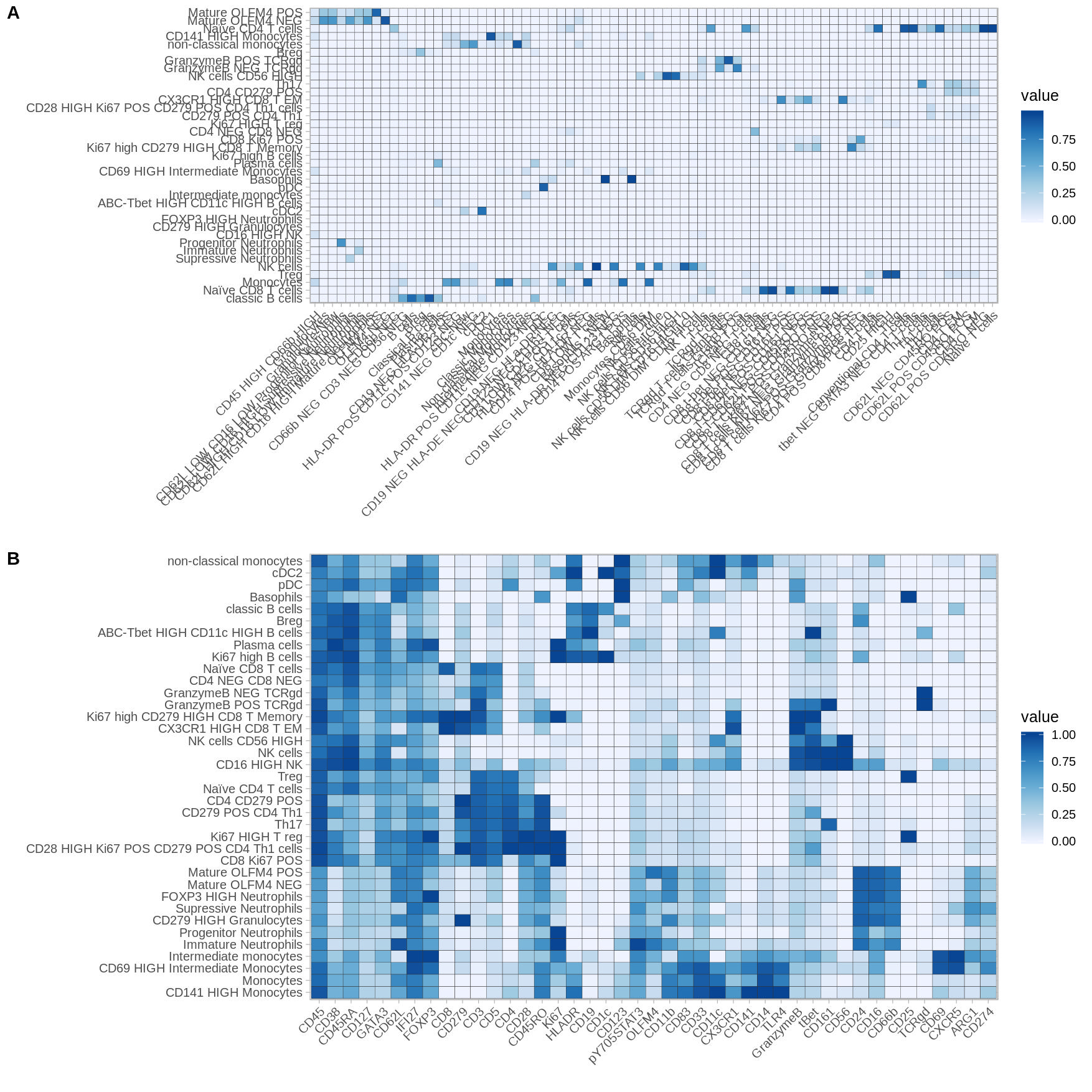

Supplement: Supplementary file 4 — Supporting Information [file CTM2-13-e1507-s003.tif]

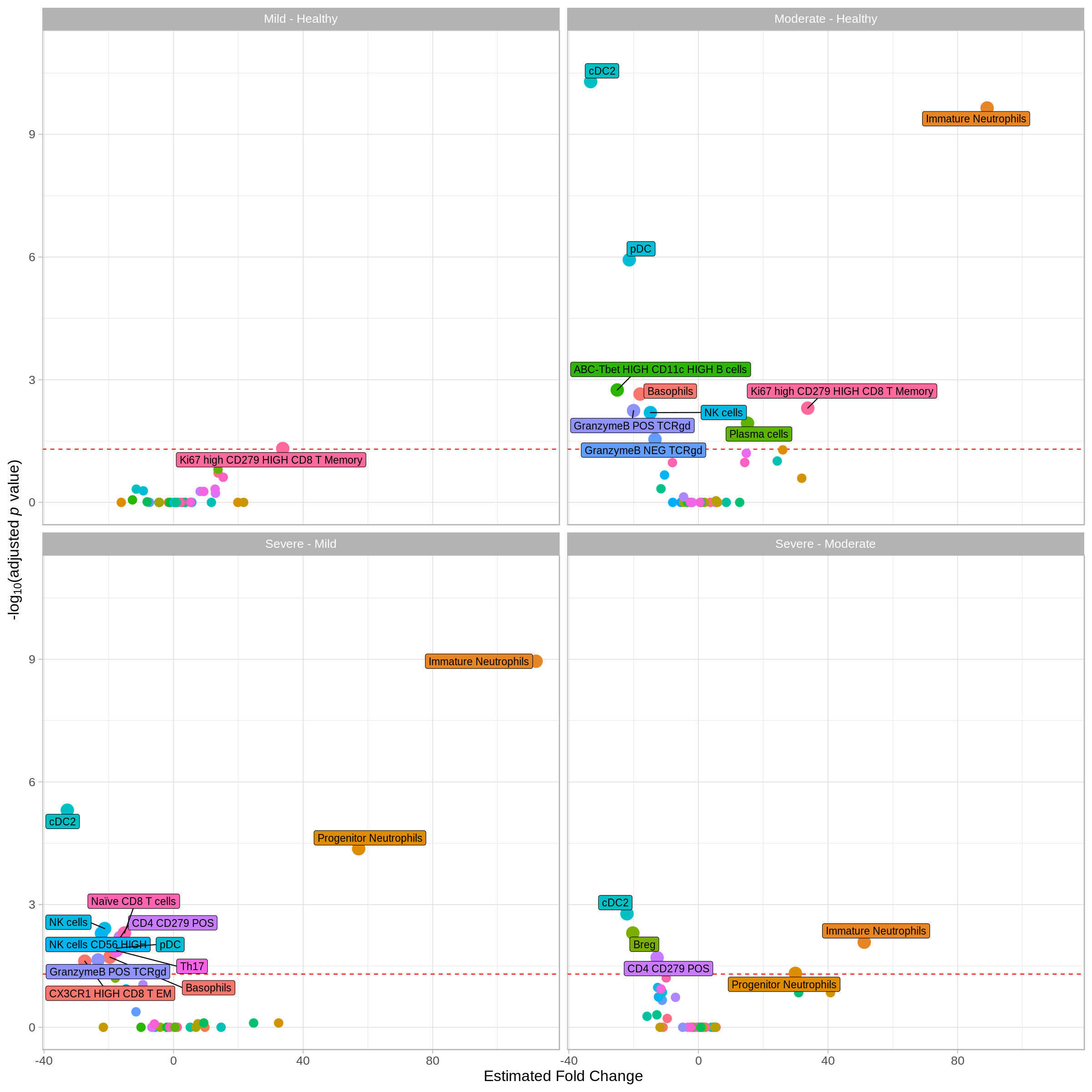

Supplement: Supplementary file 5 — Supporting Information [file CTM2-13-e1507-s004.tif]

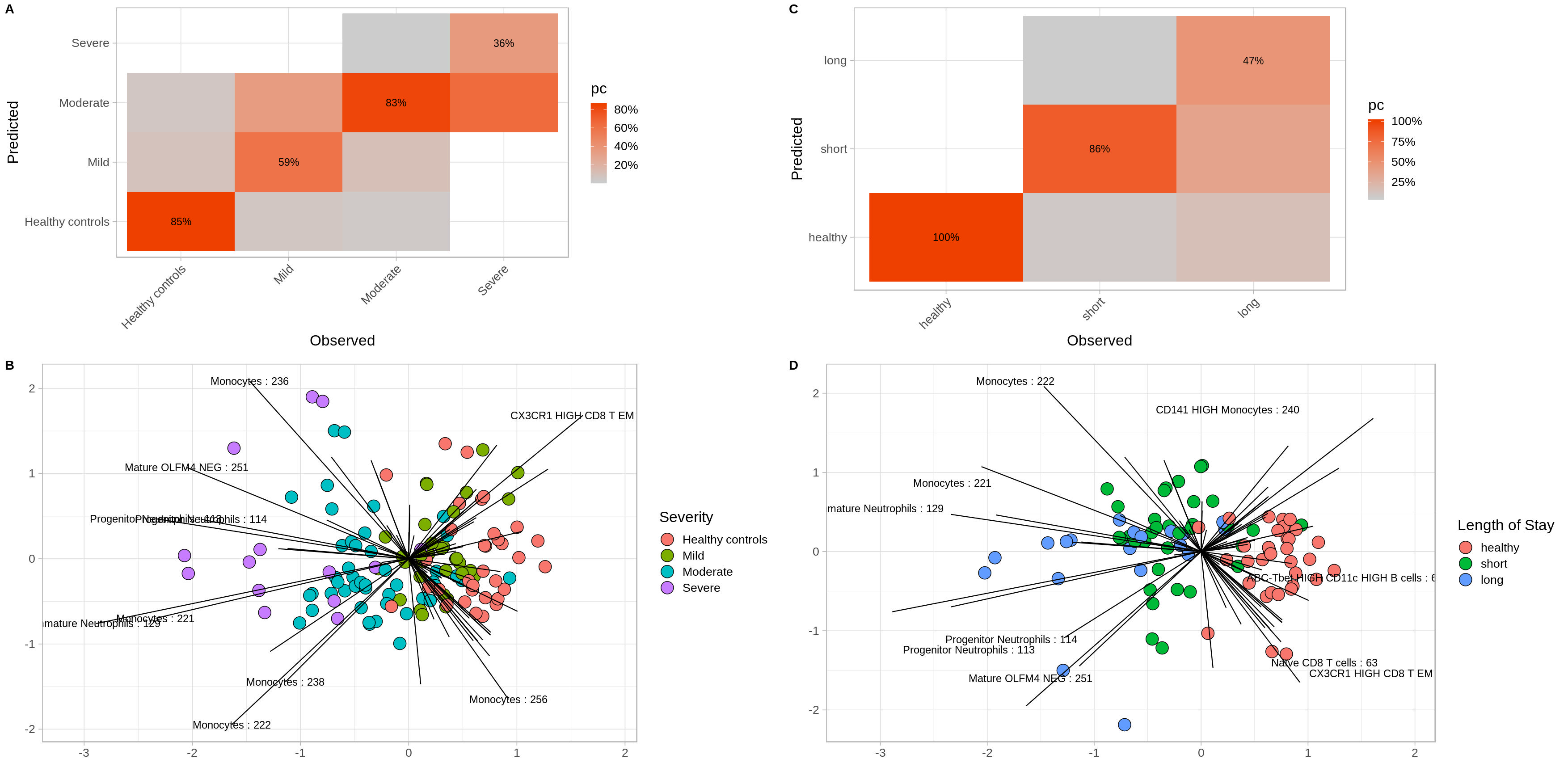

Supplement: Supplementary file 6 — Supporting Information [file CTM2-13-e1507-s005.tif]

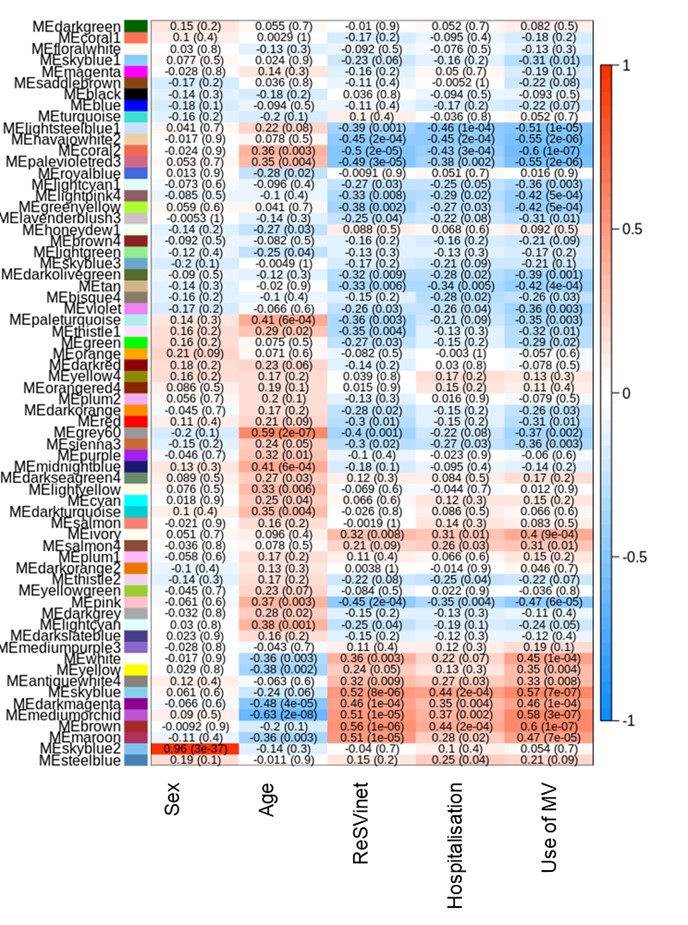

Supplement: Supplementary file 7 — Supporting Information [file CTM2-13-e1507-s009.jpg]

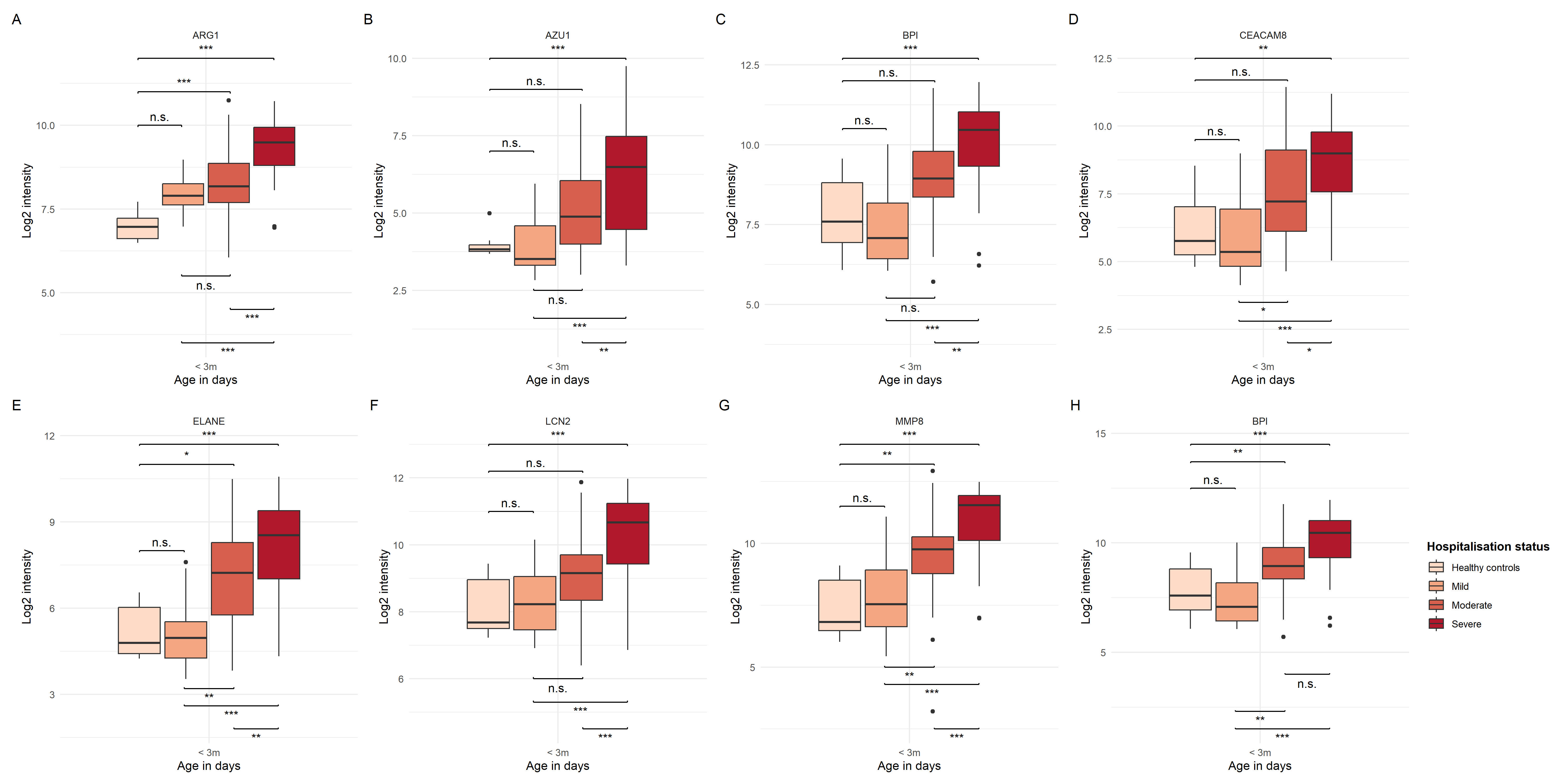

Supplement: Supplementary file 8 — Supporting Information [file CTM2-13-e1507-s008.tif]

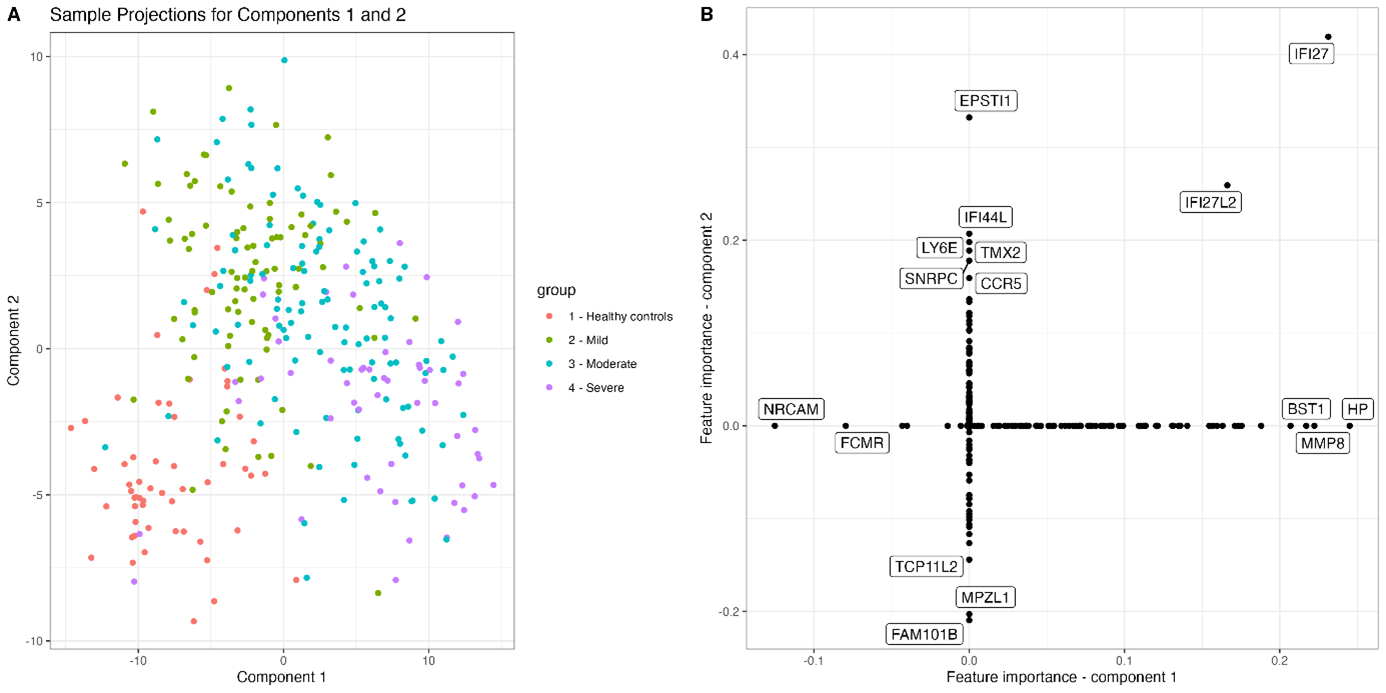

Supplement: Supplementary file 9 — Supporting Information [file CTM2-13-e1507-s007.png]
